# Forecasting the Mechanical Properties of Plastic Concrete Employing Experimental Data Using Machine Learning Algorithms: DT, MLPNN, SVM, and RF

**DOI:** 10.3390/polym14081583

**Published:** 2022-04-13

**Authors:** Afnan Nafees, Sherbaz Khan, Muhammad Faisal Javed, Raid Alrowais, Abdeliazim Mustafa Mohamed, Abdullah Mohamed, Nikolai Ivanovic Vatin

**Affiliations:** 1 Department of Civil Engineering, COMSATS University Islamabad, Abbottabad Campus, Abbottabad 22060, Pakistan; 2 NUST Institute of Civil Engineering NICE, School of Civil and Environmental Engineering SCEE, National University of Sciences and Technology NUST, Sector H-12, Islamabad 44000, Pakistan; skhan.1ms21nice@student.nust.edu.pk; 3 Department of Civil Engineering, Jouf University, Sakaka 72388, Saudi Arabia; rnalrowais@ju.edu.sa; 4 Department of Civil Engineering, College of Engineering, Prince Sattam Bin Abdulaziz University, Alkharj 16273, Saudi Arabia; a.bilal@psau.edu.sa; 5 Building & Construction Technology Department, Bayan College of Science and Technology, Khartoum 210, Sudan; 6 Research Centre, Future University in Egypt, New Cairo 11835, Egypt; mohamed.a@fue.edu.eg; 7 Institute of Civil Engineering, Peter the Great St. Petersburg Polytechnic University, 195291 St. Petersburg, Russia; vatin@mail.ru

**Keywords:** plastic concrete, irradiated plastic waste, ensemble techniques, bagging, boosting, predictive modeling, correlation coefficient, sensitivity analysis, cross-validation, machine learning

## Abstract

Increased population necessitates an expansion of infrastructure and urbanization, resulting in growth in the construction industry. A rise in population also results in an increased plastic waste, globally. Recycling plastic waste is a global concern. Utilization of plastic waste in concrete can be an optimal solution from recycling perspective in construction industry. As environmental issues continue to grow, the development of predictive machine learning models is critical. Thus, this study aims to create modelling tools for estimating the compressive and tensile strengths of plastic concrete. For predicting the strength of concrete produced with plastic waste, this research integrates machine learning algorithms (individual and ensemble techniques), including bagging and adaptive boosting by including weak learners. For predicting the mechanical properties, 80 cylinders for compressive strength and 80 cylinders for split tensile strength were casted and tested with varying percentages of irradiated plastic waste, either as of cement or fine aggregate replacement. In addition, a thorough and reliable database, including 320 compressive strength tests and 320 split tensile strength tests, was generated from existing literature. Individual, bagging and adaptive boosting models of decision tree, multilayer perceptron neural network, and support vector machines were developed and compared with modified learner model of random forest. The results implied that individual model response was enriched by utilizing bagging and boosting learners. A random forest with a modified learner algorithm provided the robust performance of the models with coefficient correlation of 0.932 for compressive strength and 0.86 for split tensile strength with the least errors. Sensitivity analyses showed that tensile strength models were least sensitive to water and coarse aggregates, while cement, silica fume, coarse aggregate, and age have a substantial effect on compressive strength models. To minimize overfitting errors and corroborate the generalized modelling result, a cross-validation K-Fold technique was used. Machine learning algorithms are used to predict mechanical properties of plastic concrete to promote sustainability in construction industry.

## 1. Introduction

With the rise in population and growing urbanization, infrastructure planning is increasing day by day. This demand has resulted in a significant increase in the construction industry’s growth. Concrete is a common building material that is composed of cement, fine aggregate, coarse aggregate, and water [1]. Because of its durability, increased strength, ease of use, and other advantageous features, concrete is widely employed as a construction material [2]. It has served as the foundation of modern existence. A rise in population can cause an increase in urbanization demand which results in high concrete demand in construction industry [3]. Humanity has been willing to overlook concrete’s environmental disadvantages for many years in exchange for concrete’s obvious advantages. Having a strong foundation is appealing in these times of rapid change, but it may also create more problems than it solves if used to its full potential.

After water, concrete is the second most commonly used substance on the earth [4]. There are numerous environmental and human health hazards associated with concrete. Carbon dioxide emissions from concrete-making accounted for 7% of worldwide carbon dioxide emissions in 2018, which is a major contributor to climate change, as it destroys the ozone layer, which raises the global temperature [5]. Calcination, the process of heating raw materials, such as limestone and clay, to temperatures greater than 1500 °C, results in the release of CO_2_ [6]. Cement production releases around 0.9 pounds of CO_2_ into the environment for every pound of cement produced [7]. Since the cement is only a fraction of the constituents in concrete, manufacturing a cubic yard of concrete is responsible for emitting about 400 lbs of CO_2_. The focus of reductions in CO_2_ emissions during cement manufacturing is on energy use, and the cement industry is striving to continuously reduce its CO_2_ contribution.

Consequently, each year, around 0.28 billion tonnes of plastic waste is produced worldwide [8]. A lot of it ends up in landfills, contaminating the environment and endangering aquatic life. Because of its durability and inexpensive cost, plastic is widely used but it has major environmental effects [9]. New ways for recycling plastic waste are being used nowadays. Plastic recycling saves 7.4 cubic yards of landfill area every tonne [10]. Recycling is on the rise because of greater environmental awareness and financial reasons. As a result of rapid industrial growth and urbanization, the amount of waste plastics produced each year has expanded uncontrolled. The environment suffers greatly as a result of this plastic waste. To reduce pollution, many products are produced from reusable waste plastics. Plastic waste is now recycled, but only a small percentage of it is recycled in comparison to manufacturing; the rest ends up in landfills, causing major environmental dangers. After being buried, plastic might take up to 1000 years to decompose. When it is burned to get rid of it, toxic chemicals like sulphur dioxide and carbon dioxide are emitted, harming both the environment and human health [11]. Plastic waste has become a major environmental concern in modern society. The increased use of numerous types of plastic objects is one of the most major environmental concerns. Plastic waste in large quantities, as well as its low biodegradability, has a negative influence on the environment [12]. To store such large influxes of plastic garbage, which demand vast tracts of land, and which cannot be recycled in their entirety, humans use a variety of plastic products throughout their daily lives. Plastic recycling is an environmentally friendly approach to limit the amount of waste burnt in landfills in the materials sector. As a result, adopting a strategy for utilizing plastic in the construction industry could be quite advantageous in the current situation. Concrete is a crucial input in the construction industry, and it is made up of cement, fine aggregates, and coarse aggregates. Due to high demand and limited supply, these basic resources are becoming increasingly difficult to obtain. As a result, using waste plastics as raw material in concrete may partially resolve both concerns. To accomplish sustainable development, plastic can be crushed and used as a concrete component, this sort of material has become a significant research topic in recent years. Some positive aspects of using plastic in concrete includes enhanced tensile and compressive strengths. Plastic in concrete also provides dense packing, thus reducing the deal weight of concrete. It also provides better resistance against chemical attack. Utilizing plastic in concrete is associated with less cement demand, resulting in less cement production. Moreover, plastic is recycled instead of being dumped in land-fills or being burnt. Hence, emission of harmful gases will be reduced.

Many laboratory tests have been conducted to examine the effect of partial replacement of concrete inputs by plastic waste on various properties of concrete. From the literature review, it is observed that using the plastic waste in concrete as an additive reduces the carbon emission but a decrease in some of the mechanical properties of concrete is also examined [13,14,15,16]. A detailed literature review is performed to discover the best grade polymer and its optimal quantities and additives for enhanced high strength [13]. We attempted to replace the fine aggregate with granulated plastic waste at different percentages. The test conducted on hardened concrete revealed a steady decrease in concrete strength as more plastic granules were added to the concrete mix [14]. The effects of replacing natural aggregate with non-biodegradable plastic aggregate made consisting of mixed plastic waste in concrete were studied. In the range of 9 to 17% at all curing ages, the decline in compressive strength (f_c_’) is mainly attributable to the weak adhesion of waste plastic to the cement paste. Compressive strength was increased by 23% when fine aggregate was replaced with irradiated plastic waste up to 5% [17]. Similarly, with a water–cement ratio of 0.52 and a water–cement ratio of 0.42, the f_c_’ increased by 8.86% and 11.97%, respectively, using fine aggregate as a 5% replacement for polyethylene terephthalate waste [15]. Similarly, it was studied that by replacing the natural coarse aggregate from 0 to 15% with waste plastic bottle caps, an increasing trend in f_c_’ was noticed but the course was reversed when the percentage was increased beyond 15% [16]. At varied mix ratios, the mechanical properties of plastic concrete display aberrant behaviour. A correlation among the properties of plastic waste and quantity of constituents used in concrete is necessary for altering this behaviour and promoting the widespread use of plastic in concrete. To achieve this, several artificial intelligence (AI) modelling techniques are utilized, as well as empirical models to support long progress. For plastic concrete design, basic mechanical parameters including f_c_’ and split tensile strength (f_sts_) should be considered.

Computational modelling approaches could be a viable alternative to the time-consuming complexity of laboratory-based mixture optimization [18]. To establish the optimal concrete mixes, these methods create objective functions from the concrete elements and their attributes and then identify the ideal concrete mixes using optimization methods. In the past, objective functions for linear and nonlinear models were designed separately. However, because of the considerably nonlinear correlations among concrete attributes and input parameters, the relations of such models cannot be established accurately. Consequently, researchers are utilizing machine learning (ML) techniques for forecasting concrete characteristics. Previously, a variety of ML techniques were utilized to forecast properties of concrete including f_c_’, elasticity modulus, and f_sts_. The most often used machine learning approaches were multi-layer perceptron neural networks (MLPNN), artificial neural networks (ANN), support vector machines (SVM), decision trees (DT), and genetic engineering programming (GEP) [19,20,21]. Researchers have used supervised machine learning and its algorithm to handle complicated issues in a variety of domains, including the prediction of concrete’s mechanical properties.

The multi-layer perceptron neural network (MLPNN) is a class of artificial neural network (ANN), which is a non-linear computer modelling method capable of establishing input–output relations for complex issues. SVM models are trained to find a global solution during training because model complexity is considered a structural risk in SVM training [22]. Classification problems are best solved using the decision tree, a supervised learning technique. In a tree-structured classifier, each leaf node represents a classification result, with internal nodes corresponding to dataset features, branching corresponding to decision rules. In the field of computational intelligence, the GEP is one of the most recent methodologies. Advanced genetic algorithms use an expression tree to express non-linear relationships. The robust architecture of deep learning (DL) algorithms, in contrast to previous ML techniques, allows researchers to better predict results. Because of the enormous amount of data collected over the past decade, DL is becoming increasingly popular. Thus, neural networks have been able to demonstrate their potential since they improve with an increasing amount of input data. When compared to typical machine learning techniques, more data will not necessarily lead to better results. When it comes to machine learning algorithms, ensemble approaches have a significant advantage over the competition [23]. Due to its capacity to tackle complicated and tough issues with exceptional precision, the DL technique has helped to promote the use of machine learning algorithms across a wide range of industries. The random forest (RF) technique was utilized to predict the mechanical properties of high-strength concrete, and statistical analyses, such as mean absolute error (MAE), relative root mean squared error (RRMSE), root mean square error (RMSE), and were employed to evaluate the models’ performance. This model outperformed all others since it relies on a weak base learner decision tree and provides a more accurate coefficient of determination (R^2^) = 0.96 [24]. A study was conducted by [20] for estimating the uniaxial f_c_’ of rubberized concrete by using RF with an optimization technique. The authors claimed that the model was accurate and had a high correlation. Moreover, f_c_’ was predicted using SVM, ANN, DT, and linear regression approaches by [25]. The DT approach was found to predict f_c_’ with minimum error and to perform better than other methods, the correlation coefficient (R^2^) was 0.86, and the best mean absolute error was 2.59 using the DT algorithm. For the purpose of forecasting the strength of self-compacting geopolymer concrete generated from raw ingredients, ANN and GEP models were developed. Using empirical relationships to predict output parameters, the authors observed that the GEP model performed better than the ANN model [26]. MLPNN, SVM, DT, and random forest (RF) algorithms were used to evaluate the performance of ensemble techniques in predicting the f_c_’ of high-performance concrete. The results suggested that using the bagging and boosting learners improved the individual model performance. In general, the random forest and decision tree with bagging models performed quite well, with R^2^ = 0.92. On average, the ensemble model in machine learning enhanced the performance of the model [24]. Ref. [25] declared that GEP and ANN are effective and efficient methodologies for estimating swell pressure and unconfined compression strength of expansive soils, according to the comparison results. The mathematical GP-based equations that were created represent the uniqueness of the GEP model and are relatively simple and efficient. The R^2^ values for both swell pressure and unconfined compression strength of expansive soils lie in the acceptable range of 0.80. In terms of accuracy, the order followed by the techniques is ANN > GEP > ANFIS. The GEP model outperformed the other two models in terms of closeness of training, validation, and testing dataset with the ideal fit slope.

An effort has been made in this study to encourage plastic waste use in concrete, and studies have been undertaken to focus on carbon footprint reduction by employing ensemble ML techniques to use plastic waste as an additive or a replacement in concrete for greater long-term sustainability. The purpose of this research is to evaluate and use ensemble learning methodologies over individual learning models to predict the strength of PC. According to the authors, there is no previous study that employs ensemble machine learning modelling for plastic concrete.

## 2. Experimental Investigation

### 2.1. Selection of Materials

Plastic debris made of high-density polyethylene (HDPE) was separated from scrap gathered at a local scrap market. HDPE is a material that is frequently utilized in the production of shampoo bottles, mobile oil canisters, and containers for storing water. Table 1 summarises the features of HDPE [27]. Gamma-radiation was used to detoxify waste plastic. It was crushed into flakes ranging in size from 2 mm to 4 mm prior to treatment. So that fine aggregate and cement could be used instead of it, it was mechanically processed. After that, the powdered waste plastic was sent to sieve analysis for quality assurance. Cement with an average particle size of 75–100 microns has been substituted for the plastic that would have gone through mesh number 200. Fine aggregate was used to fill up the gap left by the removal of the other. ASTM C150/150M specifies the use of Type-1 cement as a binding medium. In all its fresh and hardened forms, fine aggregate makes up 70% to 80% of the concrete’s volume. Lawrencepur sand from Pakistan was employed in this study, and its fineness modulus was determined to be 2.47 using sieve analysis following ASTM C136. Additionally, the specific gravity of 2.60 was determined using the pycnometer apparatus following ASTM C128. In this investigation, coarse aggregate with a fineness modulus of 7.52 and a maximum aggregate size of 19 mm was used following ASTM C33. The silica fume utilized in the research was acquired from a chemical store in Rawalpindi and met the ASTM C 1240 standard specification, containing 87% SiO_2_ and having a specific area of 15 m^2^/g.

### 2.2. Treatment Using Gamma Radiation

Treatment of gamma radiation of polymeric polymers alters their chemical and physical properties. This approach is becoming increasingly used for sterilizing medical devices and irradiating foods for preservation. Worldwide, the most often utilized source of gamma radiation is cobalt-60 (radioactive isotope) [28]. Rays are generated using electron beams with energy between 1.173 and 1.332 MeV (Mega Electron Volts) [29]. Co-60 has a half-life of approximately 5.27 years [30]. Gamma radiations as a source of Co-60 radionuclide were utilized to cure HDPE. The under-consideration plastic polymer was dosed at a rate of 50 Gray/min, resulting in a cumulative dose of 100 kGY. The 60 kg sample was divided into four 20 kg bags and packed in a plastic bag by machine to ensure it was airtight. Radiation induces crosslinking between the polymer’s chain structure and increases the polymer’s crystallinity. X-ray diffraction was used to investigate the influence of radiation. This cross-linking technique enhances the crystallinity of the polymer chain, which results in enhanced HDPE characteristics [31]. After cross-linking, the resulting HDPE offers a 20-fold increase in environmental stress crack resistance and a tenfold increase in molecular weight over standard HDPE. Additionally, the impact and f_sts_ of treated HDPE are boosted by fivefold [32].

### 2.3. Diffraction Analysis of Conventional and Irradiated Plastic Waste

**Figure 1:**
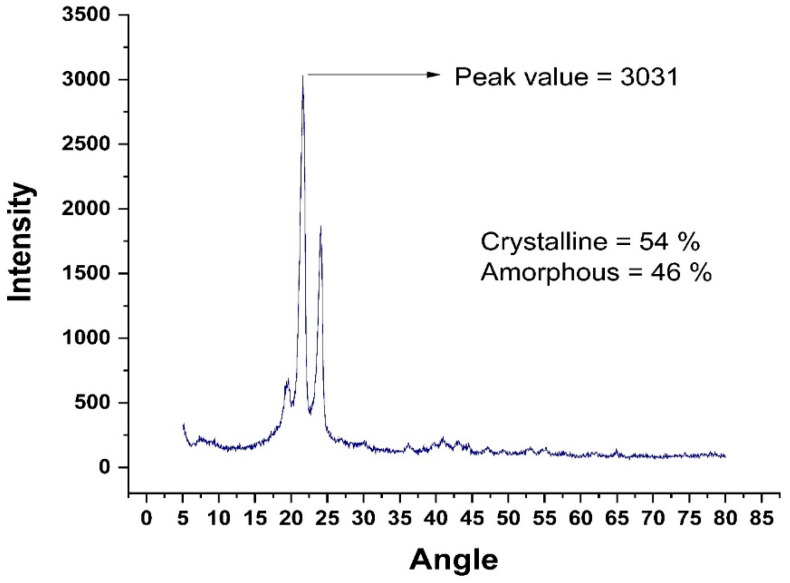
XRD Analysis of Virgin HDPE.

**Figure 2:**
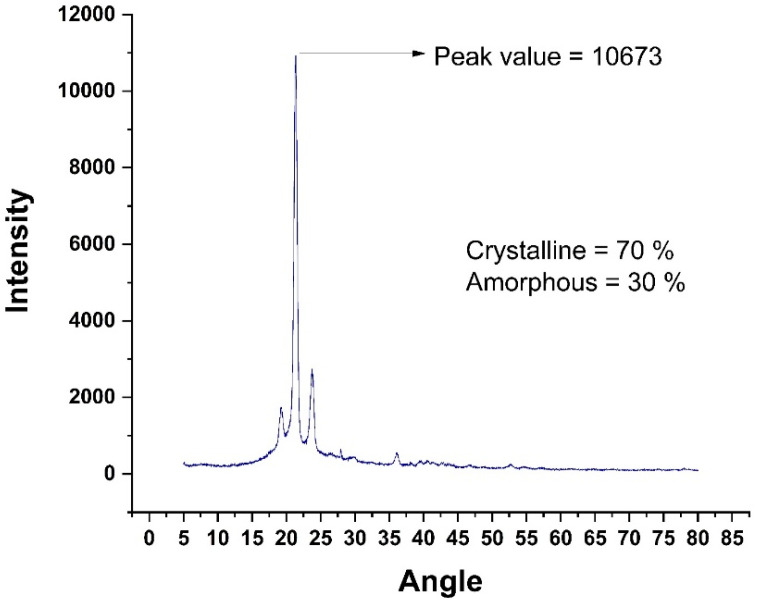
XRD Analysis of Irradiated HDPE at 100 kGY.

The X-ray diffraction (XRD) technique can be used to investigate the material’s structural information and forecast its mechanical response under various stress circumstances. XRD analysis was used to examine the effects of gamma radiation on RPW and IPW in this work. Flakes of 2–4 mm in diameter were fed into an X-ray diffractometer with a voltage of 20–40 Kev (Kilo Electron Volts). Using rays with a wavelength of 1.5418 (Angstrom), the sample spun in the apparatus, ensuring that each particle of the material was exposed to the radiation. The crystallinity of the sample was determined by plotting the intensities and rotation angle on a graph. The peak area is directly related to the degree of crystallinity. Integrating the areas of all of the curves yields a precise degree of crystallinity [33]. Gamma radiation has been shown to improve waste plastic’s mechanical qualities by enhancing its microstructure and making it crystallized. Table 2 shows the XRF analysis results for chemical composition. Figure 1 and Figure 2 refer to XRD analysis of radiated and irradiated plastic, respectively. 

### 2.4. Mix Proportioning

The constituents utilized in the investigation were readily obtainable in the area, and their attributes are listed above. Calculations for the mix design of concrete with a 3 ksi f_c_’ were conducted using water to cement ratio of 0.50 to 0.55 to ensure a three-inch slump value. This investigation employed a concrete mix mixture of 1:1.86:2.45 by mass. Two distinct mixtures were created. In the initial mix, cement was replaced with RPW and IPW in various quantities, including 1%, 2%, 3%, 5%, and 7% by weight of cement. The fine aggregate was replaced with RPW and IPW in the same quantities in the second mix. Six × twelve-inch concrete cylinders were cast and tested after three, seven, and twenty-eight days of curing.

## 3. Modelling Dataset

Experimental tests, i.e., f_c_’ test and f_sts_ test have been performed on 160 cylinders at different percentages. It has been customary for laboratories to prepare test cylinders to meet these requirements and adhere to building specifications. The trained models were put to the test using data from experiments. A model that is solely based on experimental data performs admirably, yet it cannot be called the best-performing model. The plastic concrete database containing 320 tests for each outcome, i.e., f_c_’ and f_sts_ was compiled using data from internationally published studies [14,34,35,36,37,38,39,40,41,42,43,44,45,46]. Table 3 and Table 4 illustrate the maximum and minimum ranges of input parameters that are functions of outputs. Table 5 and Table 6 also includes the frequency distribution and statistical description of the measure, including the mean, standard deviation, median, and skewness. Prior to building a model, the input factors affecting the mechanical characteristics of plastic concrete must be chosen. The major constituents impacting concrete’s characteristics are identified and a generalized function is devised. The function is defined as follows:

f_c_, f_st_ (MPa) = (C, FA, CA, W, SF, PW, SP, Age)

**Figure 3:**
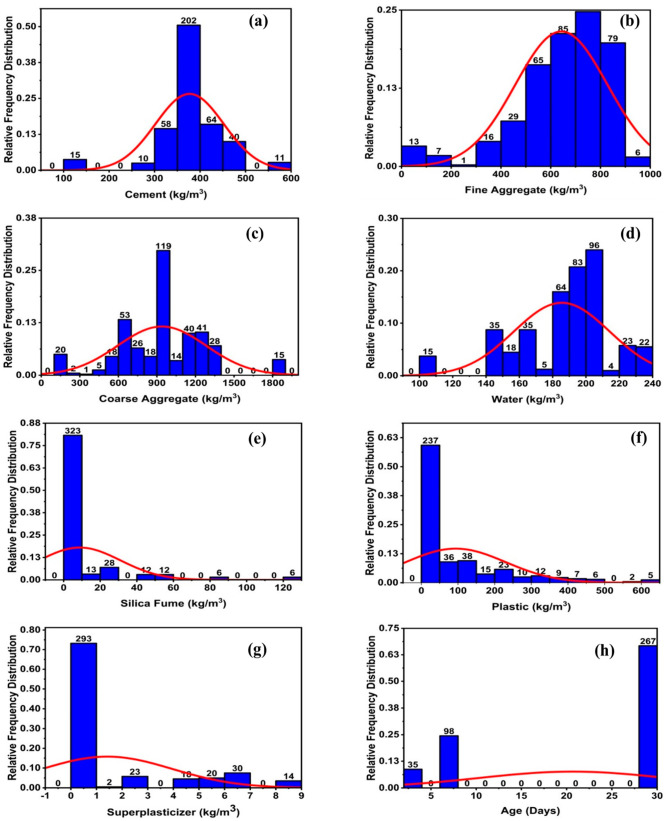
Compressive strength parameters’ relative frequency distribution; (**a**) cement, (**b**) fine aggregate, (**c**) coarse aggregate, (**d**) water, (**e**) silica fume, (**f**) plastic, (**g**) superplasticizer, and (**h**) age.

**Figure 4:**
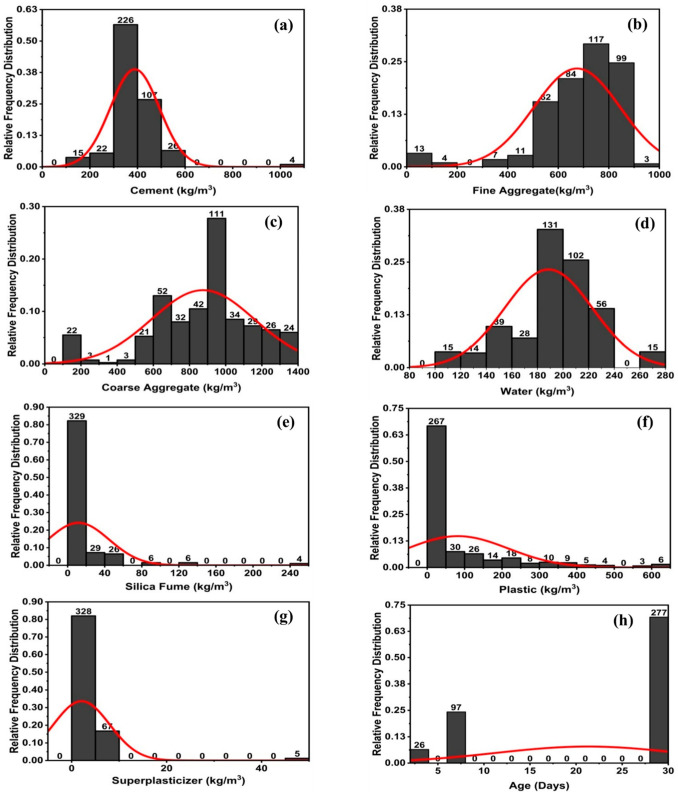
Split tensile strength parameters’ relative frequency distribution; (**a**) cement, (**b**) fine aggregate, (**c**) coarse aggregate, (**d**) water, (**e**) silica fume, (**f**) plastic, (**g**) superplasticizer, and (**h**) age.

The model’s performance is strongly impacted by its variables. The correlations between their distributions and the input factors affecting their compressive and tensile strength are shown in Figure 3 and Figure 4, respectively. It illustrates the link between the data points by including the relative frequency distribution with them. Additionally, it assists us in doing statistical analysis by displaying the database, as seen in Figure 3 and Figure 4.

## 4. Methodology

### 4.1. Machine Learning as an Approach to Artificial Intelligence

Artificial intelligence (AI) is proven to be a more efficient modelling technique than more conventional ways. AI offers several benefits when it comes to dealing with unclear challenges and is an effective tool for resolving such complex scenarios. When testing is not feasible, AI-based solutions are a viable choice for identifying engineering design parameters, resulting in considerable time and effort savings for human testers. Additionally, AI can accelerate decision-making, reduce mistake rates, and increase computing efficiency [47]. There has been a surge in interest in the application of AI to all disciplines of engineering in recent years, igniting a variety of objectives and dreams. The civil engineering community has witnessed a substantial surge in the use of various AI branches/methods throughout its many areas. ML, pattern recognition (PR), and deep learning (DL) are three techniques to artificial intelligence that have lately garnered considerable interest and are establishing themselves as a new class of intelligent structural engineering solutions. ML is a rapidly growing area of artificial intelligence AI, and it is generally used in the construction industry for predicting material behaviour. The purpose of this study is to examine how social variables are included In multi-criteria infrastructure evaluation approaches. By including social elements into the evaluation of infrastructure sustainability through the use of multi-criteria assessment methodologies [48]. Comprehensive research of evolutionary computation, a subfield of artificial intelligence, was undertaken in the context of structural design [49]. Similarly, cutting-edge techniques in civil engineering, construction, and building technology were surveyed to make environmentally friendly solutions [50]. Ref. [51] studied applications of AI in geotechnical engineering. A survey was conducted to see how high-rise building optimization was progressing [52]. The research was conducted to synthesize ideas in the emerging direction of AI applications in civil engineering. A wide range of techniques are included in this list: evolutionary computation (EC), neural networks (NN), fuzzy systems (FS), expert systems (ES), reasoning (RS), classification (CL), and learning (CL), among others [53].

**Figure 5:**
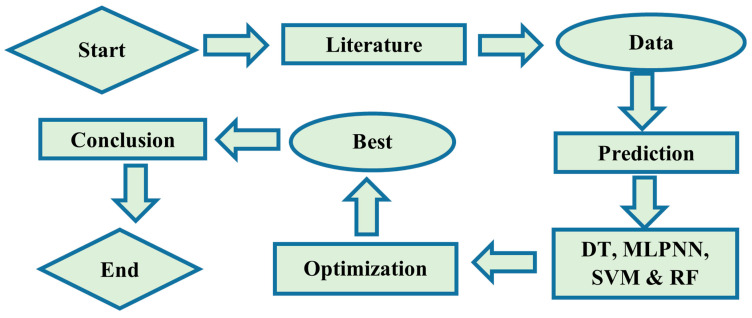
Flow chart of ML techniques.

Although the cited review articles addressed AI applications in civil engineering, they primarily focused on old methodologies and did not address newer methods having ensemble algorithms. This study is employed to use machine learning techniques, such as DT, MLPNN, SVM, and RF to estimate the f_c_’ and f_sts_ of plastic concrete as depicted in Figure 5. These algorithms are considered best for data prediction, and the choice of selection of these techniques was based on their extensive use in relevant studies. Furthermore, ensemble learners are used to predicting the modelling strength of concrete. In terms of computing performance and time required for processing, ML models are quite important. In comparison with standard models, the error rate is almost non-existent. In this research, a comparative study is drawn between individual models and ensemble models. The next section provides a quick overview of the various modelling approaches used in this research.

To begin, DT, MLPNN SVM, and RF were used to estimate the f_c_’ and f_sts_ of plastic concrete as a single separate model. After that, for each outcome new ensemble learning algorithms techniques, such as boosting, bagging, and modified learner are used for forecasting. The individual model is compared with each other and was then compared to ensemble approaches. The outcomes indicate that newly designed ensemble techniques outperform the typical individual machine learning model [54]. ML ensemble approaches were evaluated for prediction accuracy using a variety of statistical markers. Indeed, recent prediction modelling research has found that ensemble approaches are gaining favour, as they typically generate better findings than individual base learners [54].

#### 4.1.1. Decision Tree

**Figure 6:**
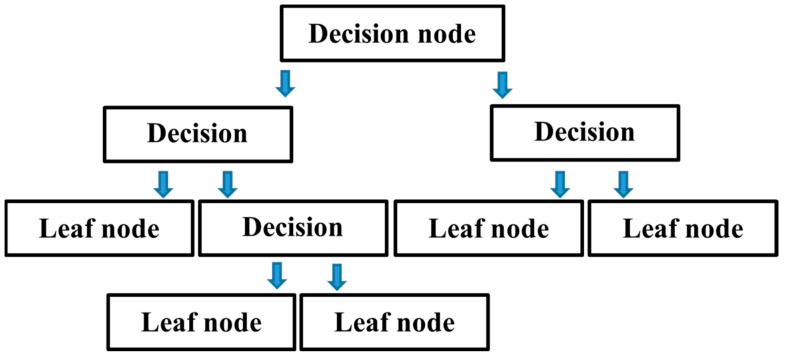
Flow chart of decision tree.

For regression and classification issues, DT is a method for predictive modelling that is used in AI. DT is a categorization scheme comprised of a series of if-else expressions. As seen in Figure 6, it is made up of several nodes, also known as leaves. Each leaf is subjected to a test, which sends a query to the node’s branches. The query will be looped until it reaches the terminal leaf. Each leaf node is correlated with the value returned as the tree’s contribution. This should be performed to construct the shortest feasible tree by prioritizing the most essential qualities. Division of learners after the first attribute, other learners come to be DT problems in their own right, although with fewer samples and fewer attributes. The complexity can be solved by using subtrees with fewer but more important properties. A node with a larger number of samples has a higher level of complexity. The complexity of a homogenous node is reduced because it has a sample of only one type. The objective is to create trees by repeatedly reducing the sample’s classes to achieve as pure leaf nodes as possible [55].

#### 4.1.2. Multilayer Perceptron Neural Network (MLPNN)

**Figure 7:**
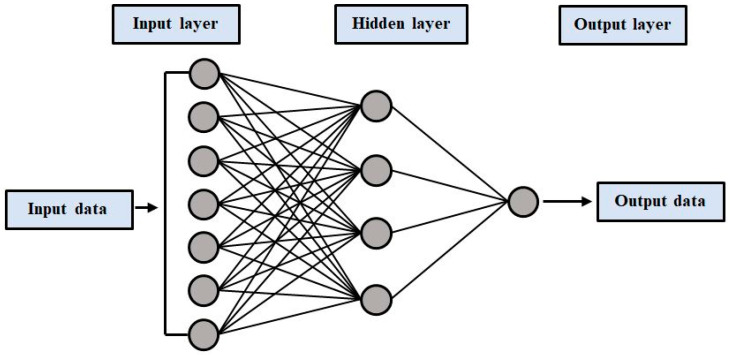
Typical neural network architecture.

One of the most effective machine learning models is the ANN model. It has been frequently used in environmental, hydrological, and engineering investigations due to its potential to address nonlinear problems. The multi-layer perceptron ANN is the most extensively utilized of the many types of ANN models (MLPNN). The MLPNN model’s basic architecture consists of three layers: the input layer, one or more hidden layers, and the output layer. Purelin, logsig, and tansig are three typical activation functions. Weights, bias, and activations functions are the most significant components in the hidden and output layers. Model training determines the weights or model parameters. All of the hidden layers employed the tansig activation function, but the output layer used purelin. Fivefold cross-validation was used to find the best structure. The best ANN model was found to have three hidden layers, with the optimal number of neurons for each hidden layer being 9, 3, and 2, respectively [56]. Figure 7 shows a typical neural network. These networks are composed of three stages: the forward-pass transmits the input and multiplies it by weight, and the model output is predicted. The anticipated outputs are compared to the provided inputs. The output of the model predicts the results by considering the input parameters into account. Depending on our performance and objectives, we employ a variety of loss functions. Backward propagation generates partial-derivatives of the cost-function relating to the individual parameters back into the function. Back propagate loss and update the model’s weights using gradient descent during this process.

#### 4.1.3. Support Vector Machine

**Figure 8:**
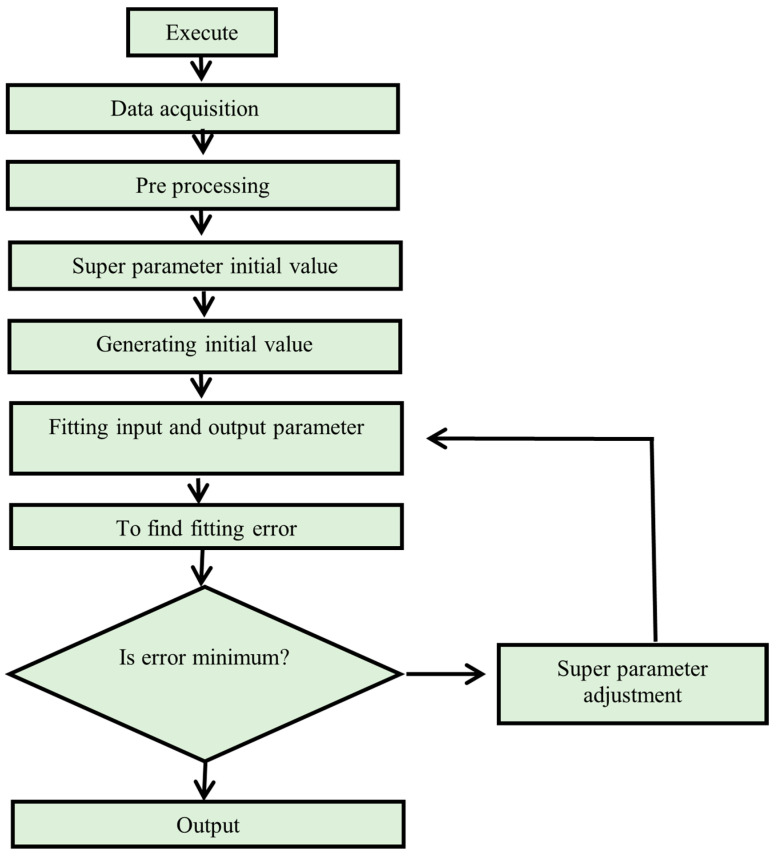
Flow chart of support vector machine.

SVM is an input–output mapping supervised learning method provided by the dataset. Classification and regression problems are solved using SVM models. SVM, on the other hand, is mostly used to solve classification difficulties. In SVM, classification is accomplished by using a hyperplane to distinguish between two classes. Each data point is represented as an n-dimensional space point where *n* is the number of features you have with the value of each feature being the coordinate value. Following that, categorization is completed by analysing the hyper-plane, which clearly distinguishes the two classes [57]. Figure 8 illustrates the SVM flow chart.

#### 4.1.4. Random Forest

Random forest regression is a supervised learning regression technique that makes use of the ensemble learning method. Random forest is a bagging technique, not a boosting technique. In random forests, trees grow parallel to one another. There is no contact between the trees throughout their construction. It works by training a large number of decision trees and then determining the class that corresponds to the mode of the classes or mean forecast of each tree. The random forest algorithm was developed in 2001 [58].

**Figure 9:**
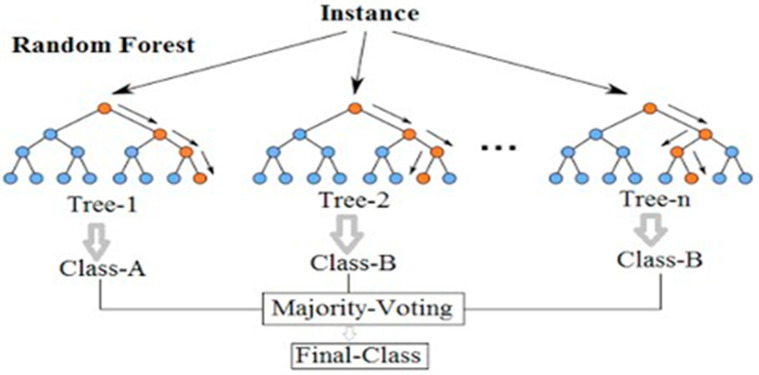
Flow chart of support vector machine.

The RF method consists of three main steps. It is used to gather trained regression trees using the training set, then to calculate the average of each regression tree’s output, and, finally, to perform cross-validation on the predicted data using the validation set [19]. A recent study by [59] concluded about the random forest that in RF the average of the learners on the nodes and the mean square error (MSE) produced among each learner were determined at each branching of the regression tree. The regression tree will stop growing if the minimal leaf node MSE is used as a branching condition until no more features are available or the total MSE is optimal. The number of regression trees and the number of random variables of nodes are two crucial custom factors. Figure 9 depicts how RF works based on classes and trees.

### 4.2. Techniques for Ensembles including Bagging and Bossing

The idea behind ensemble approaches is that by merging multiple models, you can create a far more powerful model. Multiple models, sometimes known as weak learners, are taught to tackle the same problem and then combined to obtain better results. A strong model can be created by merging all of the weak learners in a perfect configuration. Machine learning approaches use ensemble techniques to improve their recognition and prediction accuracy. By integrating and aggregating numerous weak predictive models, these methods can often assist to decrease the overfitting problems of the training set. Bagging is a subclass of ensemble modelling that is taught to minimize prediction variance by generating new data for training from a dataset using combinations and repetitions to build multiple sets of the original data. The final outcome is obtained by averaging the outputs of the component models. Boosting, like bagging, adjusts the weight of an observation based on the previous categorization. Boosting generates generally accurate prediction models. Base learners, such as MLPNNs, SVMs, decision trees, and random forests are combined with boosting and bagging in this study to predict the strength of typical concrete.

#### Ensemble Learner Parameter Calibration

**Figure 10:**
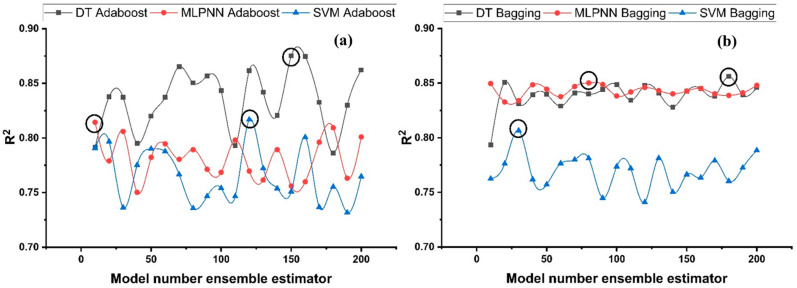
R^2^ for compressive strength ensemble models with different sub-models; (**a**) Adaboost; (**b**) Bagging.

**Figure 11:**
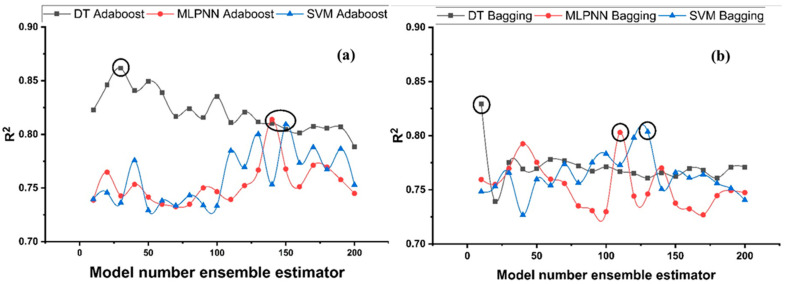
R^2^ for tensile strength ensemble models with the different sub-models; (**a**) Adaboost; (**b**) Bagging.

Features linked to the total number of sample learners and the rate at which they learn, as well as other comparable parameters that uniquely affect ensemble algorithms, may be represented using tuning parameters utilized in ensemble processes. To find the optimal sub-model range, bagging and boosting ensemble models (20 each) with 10, 20, 30, …, 200 component sub-models were generated for each base learner, and the best structures were picked based on high correlation coefficient values. Figure 10 depicts the relation among ensemble model performance and the different sub-models for f_c_’, whereas Figure 11 illustrates the relationship for f_sts_. As seen in Figure 10, the ensemble model with Adaboost generates a high value of R^2^ in the prediction aspect, where DT prevails over other boosting models. Ensemble model with highest R^2^ is selected for each ML technique.

## 5. Model Evaluation Criteria

A model’s performance for training or testing dataset may be evaluated using statistical errors, such as MAE, R^2^, RMSLE, and RMSE. Among them, the R^2^ value is considered an excellent parameter for model evaluation since it is the most accurate. The models are evaluated in this study utilizing statistical study and error measures. When combined, these metrics may offer you a wealth of information about the problems in your design. The coefficient of determination is used in this study to demonstrate the accuracy and validity of the model in question. When the model produces good results, the R^2^ value should be between 0.65 and 0.75; when the model produces poor results, the R^2^ value should be less than 0.50. The value of R^2^ may be calculated with the help of Equation (1).

(1) $$R^{2}=\frac{\sum_{i=1}^{n}\left(M_{i}-{\bar{M}}_{i}\right)(P_{i}-{\bar{P}}_{i})}{\sqrt{\sum_{i=1}^{n}{\left(M_{i}-{\bar{M}}_{i}\right)}^{2}\sum_{i=1}^{n}{\left(P_{i}-{\bar{P}}_{i}\right)}^{2}}}$$

MAE stands for average absolute error; it is the difference between predicted and observed values when all input entities are equally weighted. The absolute value is used to eliminate the negative sign. Absolute error size is determined, and the units are identical to the output units. A model with an MAE value inside a range can occasionally have extraordinarily high errors. Equation (2) is used to compute it.

(2) $$\mathrm{MAE}=\frac{1}{n}\sum_{i=1}^{n}\left|P_{i}-M_{i}\right|\;$$

RMSLE considers the relative inaccuracy between the anticipated and actual values. It is the difference between the logarithm of the predicted and actual values. RMSLE is computed as follows, where yi represents the anticipated value and yj represents the actual value.

(3) $$\mathrm{RMSLE}\;=\sqrt{\frac{1}{N}\sum_{i=1}^{N}{\left(\log\left(\mathrm{yi}+1\right)-\log\left(\mathrm{yj}+1\right)\right)}^{2}}\;$$

RMSE is the square-root of the mean of the squared discrepancies between predicted and actual measurement. It determines the mean square value of the error. It shows standard deviation of the anticipated inaccuracy. The root mean square error is a statistic that indicates the average prediction error of model while anticipating the results. The model is more accurate if the root mean square error is less. The model’s ability to effectively forecast the data is reflected by the RMSE score of 0.5. The following Equation (4) can be used to determine the RMSE.

(4) $$\mathrm{RMSE}=\sqrt{\frac{\sum_{i=1}^{n}{\left(P_{i}-M_{i}\right)}^{2}}{N}}$$

## 6. Model Outcomes for Compressive Strength and Tensile Strength

### 6.1. Decision Tree Outcomes

**Figure 12:**
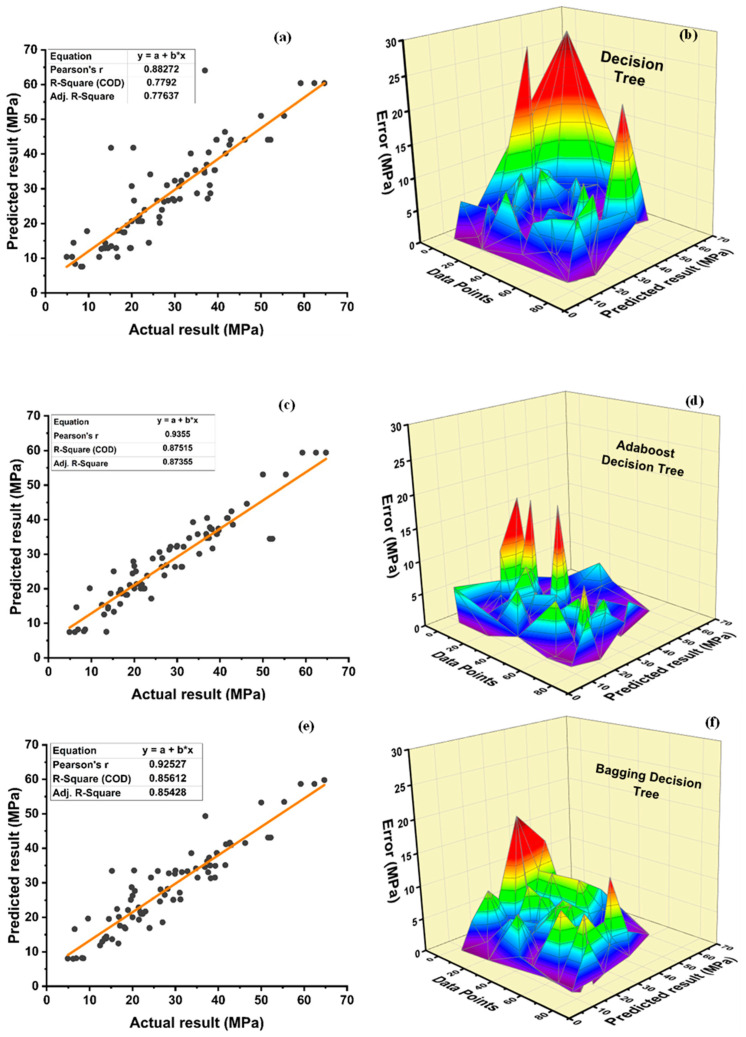
Compressive strength: (**a**) DT regression model; (**b**) DT model error distribution; (**c**) DT-adaboost model; (**d**) DT-adaboost model error distribution; (**e**) DT-bagging regression model; and (**f**) DT-bagging model error distribution.

**Figure 13:**
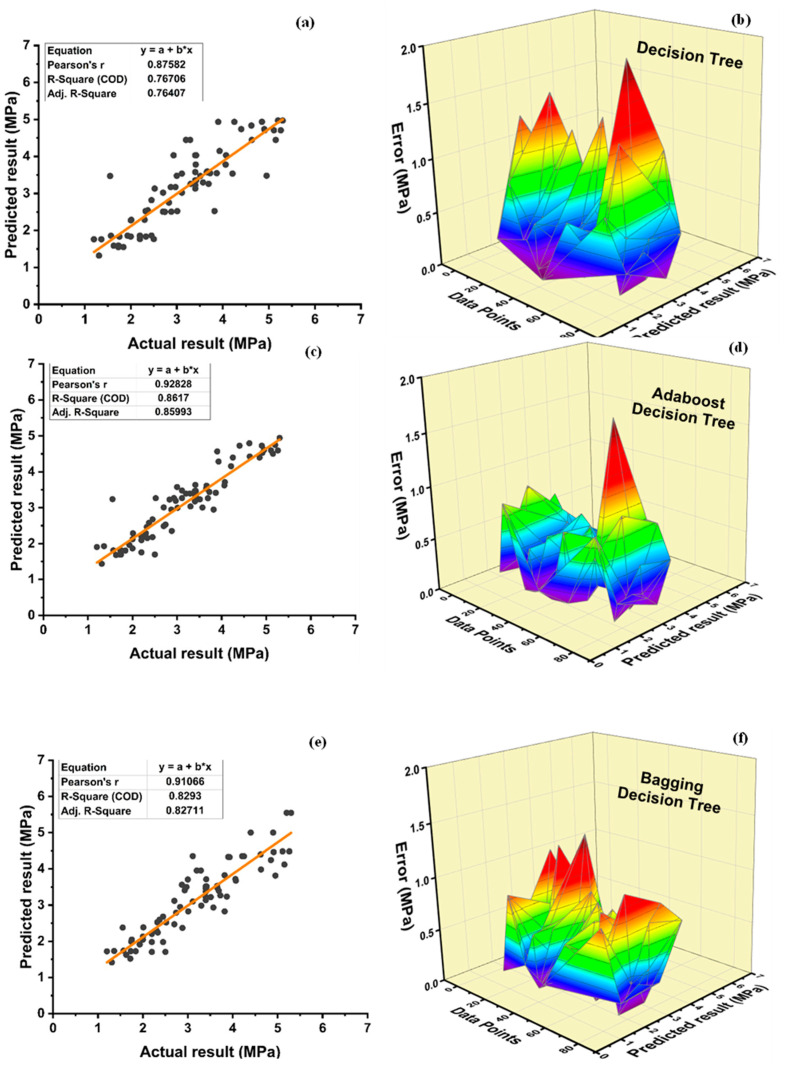
Tensile strength: (**a**) DT regression model; (**b**) DT model error distribution; (**c**) DT-adaboost model; (**d**) DT-adaboost model error distribution; (**e**) DT-bagging regression model; and (**f**) DT-bagging model error distribution.

The accuracy of nonlinear regression or supervised learning predictions is rather high, as seen in Figure 12. DT individual model provides precise and reliable performance with R^2^ = 0.779 as seen in Figure 12a. However, as seen in Figure 12c and Figure 13e, respectively, the ensemble model with boosting and bagging outperforms the individual model. Table 7 shows the comparison of individual algorithms and ensemble algorithms for all models. Table 8 shows error ranges for statistical parameters. The ensemble model’s resilient performance can also be connected to its error distribution, as seen in Figure 12d,f. As shown in Figure 12b, an average error of 4.32 MPa is observed with the maximum error of 27.09 MPa and minimum error of 0.0088 MPa, for the individual algorithm using DT approach. These attributes are enhanced using an ensemble algorithm to average, maximum, and minimum error of 3.39 MPa, 17.71 MPa, and 0.04 MPa, respectively, for a DT-adaBoost ensemble model as seen in Figure 12d and 3.87 MPa, 18.23 MPa, and 0.0057 MPa, respectively, for bagging ensemble DT model, as shown in Figure 12f. Furthermore, 70% of the data of individual DT models indicate an imprecision under 5 MPa, whereas 23.75 and 2.5% of the data indicates an error from 5 to 10 MPa and 10 to 15 MPa, respectively. Furthermore, no error is observed in the range of 15 to 20 MPa, while 3.75% of the error lies in between 20 to 30 MPa. Contrarily, the ensemble DT-adaboost algorithm’s data demonstrates no error beyond 20 MPa. Data from the ensemble DT-adaboost model displays that the error is 80% under 5 MPa, whereas 15 and 1.25% of the error is from 5 to 10 MPa and 10 to 15 MPa, respectively. Only 3.75% of the data displays that error lie from 15 to 20 MPa. Similarly, the ensemble bagging DT model for f_c_’ shows no error above 20 MPa. Data from the bagging DT model depicts an error of 70% under 5 MPa, 23.75% from 5 to 10 MPa, and 5% from 10 to 15 MPa, respectively. Only 1.25% error from bagging DT model data illustrates the error in the range of 15–20 MPa.

Figure 13a,c,e depicts the model performance versus actual f_sts_ results, while Figure 13b,d,f depicts the error between actual and predicted values. Boosting ensemble algorithm improved individual DT model of f_sts_ from R^2^ = 0.767 to R^2^ = 0.862. The average, maximum and minimum values of individual DT models for f_sts_ are 0.4 MPa, 1.92 MPa, and 0.013 MPa, respectively. The average, maximum, and minimum values are observed to be 0.30 MPa, 1.69 MPa, and 0.002 MPa, respectively, for the best ensemble DT-adaboost model and 0.35 MPa, 1.25 MPa, and 0.001 MPa for best ensemble DT bagging model. This shows an enhancement of 25, 12, and 84.62% in average, maximum, and minimum errors, respectively, using adaboost ensemble algorithm for f_sts_. Similarly, there is an improvement of 12.5, 34.9, and 92.3% in average, maximum, and minimum errors, respectively, using the bagging ensemble algorithm for f_sts_. Moreover, individual DT model data shows an error of 90% below 1 MPa, and 10% error between 1–2 MPa. Similarly, DT-adaboost model data illustrates an error of 98.75% below 1 MPa and 1.25% error between 1 and 2 MPa. For the bagging ensemble DT model, data depicts an error of 96.25 below 1 MPa, and 3.75 between 1 and 2 MPa.

The DT boosting ensemble model in comparison to the individual model improves R^2^ by 12.32% for f_c_’ and 12.39% for f_sts_. Similarly, DT-bagging ensemble model enhances R^2^ by 9.88% for f_c_’ and 8.03% for f_sts_. The values of the DT metrics are acceptable, and this approach may be used to reliably forecast the f_c_’ of the model. A model’s accuracy is greatly reliant on the number of databases used. This model consists of 400 databases for the prediction of f_c_’ and f_sts_. Table 9 and Table 10 show the statistical description for DT f_c_’ and f_sts_ models, respectively. The average, minimum, and maximum compressive strength obtained from individual DT model are 28.37 MPa, 7.59 MPa, and 64.09 MPa, respectively, as depicted in Table 9. DT-adaboost compressive strength model shows a deviation of 1.37, 1.85, and 7.27% in average, minimum, and maximum values, respectively. Similarly, the average, minimum, and maximum values deviated by 0.4, 5.14, and 6.71% for DT-bagging model when compared with individual DT compressive strength model. Table 10 shows an average value of 3.14 MPa, with 1.32 MPa, and 4.97 MPa as minimum and maximum value for DT split tensile strength model. A variance of 0.64, 9.1, and 0.61% is observed in average, minimum, and maximum values of DT adaboost split tensile strength model when compared to individual DT model. DT bagging model shows a deflection of 0, 6.82, and 11.67% in average, minimum, and maximum values, respectively, when compared with the individual split tensile strength DT model.

### 6.2. MLPNN Outcomes

**Figure 14:**
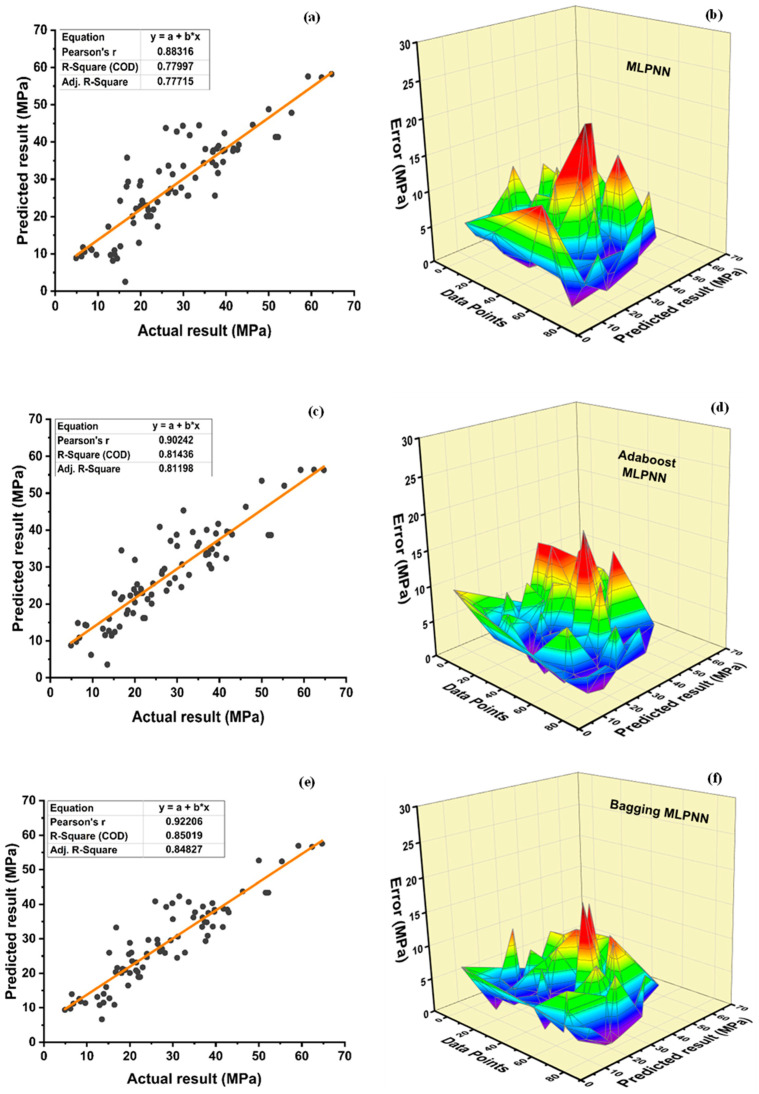
Compressive strength; (**a**) MLPNN regression model; (**b**) MLPNN regression model error distribution; (**c**) MLPNN-Adaboost regression; (**d**) MLPNN-Adaboost regression model error distribution; (**e**) MLPNN-Bagging regression model; (**f**) MLPNN-Bagging regression model error distribution.

**Figure 15:**
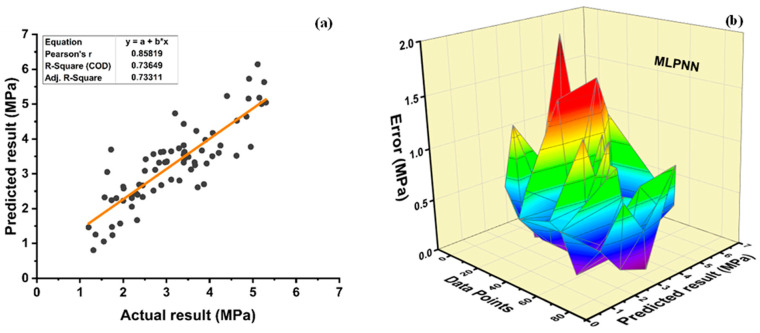

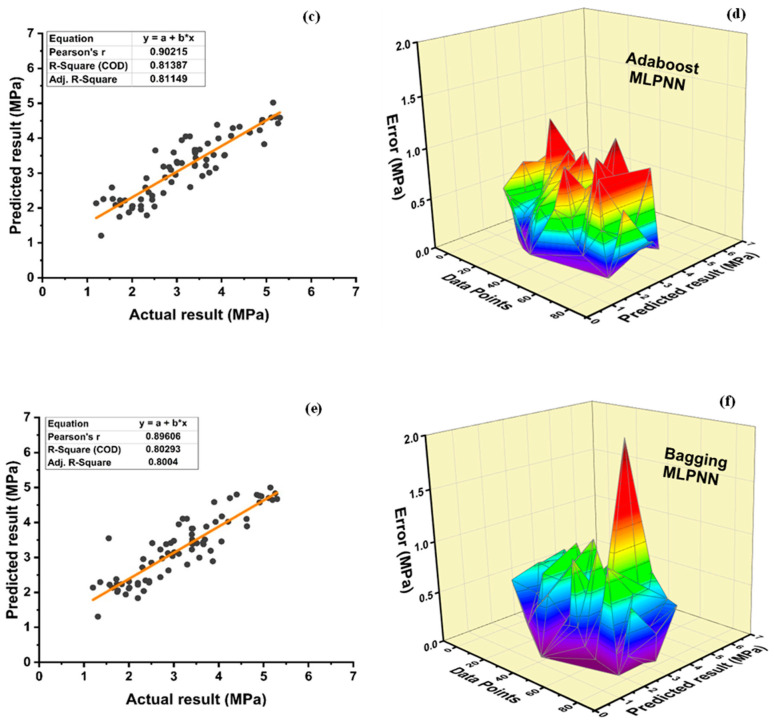
Tensile strength (**a**) MLPNN regression model; (**b**) MLPNN regression model error distribution; (**c**) MLPNN-Adaboost regression; (**d**) MLPNN-Adaboost regression model error distribution; (**e**) MLPNN-Bagging regression model; (**f**) MLPNN-Bagging regression model error distribution.

MLPNN is modelled in the same way as the decision tree, adopting ensemble learner’s methods, as shown in Figure 14. Figure 14a illustrates the relation among predicted and experimental values for individual MLPNN model of plastic concrete having R^2^ = 0.78, along with its error-distribution, illustrated in Figure 14b. Best boosting and bagging ensemble algorithm MLPNN sub-model is selected, analysed, and compared with individual MLPNN model. The individual MLPNN model demonstrates a maximum error of 18.97 MPa and minimum error of 0.029 MPa, indicating an average error of 4.89 MPa. Furthermore, data illustrates error of 63.75% below 5 MPa, 20% from 5 to 10 MPa, 13.75% from 10 to 15 MPa, and 2.5% from 15 to 20 MPa. Moreover, this model is enhanced by an ensemble adaboost algorithm that depicts R^2^ of about 0.81, as illustrated in Figure 15c. Additionally, Figure 14d shows an average, maximum and minimum values of error of 4.61 MPa, 17.66 MPa, and 0.07 MPa, respectively, for MLPNN best ensemble adaboost f_c_’ model. Moreover, 68.75% of the data depict error below 5 MPa, 22.5% from 5 to 10 MPa, 7.5% from 10 to 15 MPa, and 1.25% from 15 to 20 MPa. The bagging algorithm enhanced f_c_’ MLPNN model with R^2^ = 0.85. Figure 14f for bagging ensemble MLPNN model illustrates an average, maximum, and minimum values of error of 4.17 MPa, 16.45 MPa, and 0.04 MPa, respectively. Furthermore, 66.25% of the data show the error below 5 MPa, 26.25% from 5 to 10 MPa, 6.25% from 10 to 15 MPa, and 1.25% from 15 to 20 MPa.

The value of R^2^ is found to be 0.736 for the individual MLPNN model, as seen in Figure 15a. Boosting and bagging ensemble f_sts_ model improved R^2^ to 0.814 and 0.803, as shown in Figure 15c and Figure 15e, respectively. Figure 15b,d,f displays error distribution of f_sts_ MLPNN individual and ensemble models. An average error of 0.46 MPa is observed for individual f_sts_ MLPNN model, with 1.98 MPa and 0.012 MPa as the maximum and minimum error, respectively. Moreover, data depicted errors of 88.75% below 1 MPa, and 11.25% error between 1 and 2 MPa, respectively, whereas these statistics are enhanced in the ensemble MLPNN f_sts_ models. The ensemble boosting model depicts that an average error of 0.39 MPa is observed with an improvement of 15.22%. Maximum and minimum errors are enhanced by 42.93 and 50%, respectively, with values of 1.13 and 0.006, respectively. Moreover, adaboost MLPNN f_sts_ model data show that 96.25% of error is less than 1 MPa, and 3.75% of the error is from 1 to 2 MPa. The bagging ensemble model enhances the average, maximum, and minimum error values by 19.56, 0, and 98.08%, respectively, with the value of 0.37 MPa,1.98 MPa, and 0.00023 MPa for average, maximum, and minimum errors, respectively. Furthermore, the bagging ensemble model for f_sts_ data shows an error of 98.75% is less than 1 MPa and just 1.25 of the error is from 1–2 MPa.

MLPNN boosting ensemble technique shows an enhancement of 4.36% for f_c_’ and 10.6% for f_sts_ in terms of R^2^ when compared with the individual MLPNN model. Similarly, the bagging ensemble technique depicts an improvement of 8.97% and 9.1% for f_c_’ and f_sts_, respectively. The MLPNN can accurately forecast the compressive and f_sts_ based on the following statistics. Table 11 and Table 12 show statistical values for testing used in MLPNN models of f_c_’ and f_sts_, respectively. Individual MLPNN models yield an average compressive strength of 28.88 MPa, a minimum of 2.50 MPa, and a maximum of 58.23 MPa, as shown in Table 11. Compressive strength of the MLPNN model exhibits a variance of 1.98%, 43.68%, and 3.25% in the average, minimum, and maximum values accordingly. Similarly, the average, maximum, and minimum values for the MLPNN-bagging model differed from the individual MLPNN compressive strength model by 0.1%, 5.14%, and 1.66 times, respectively. MLPNN split tensile strength model has an average value of 3.29 MPa, with 0.81 MPA as the minimum and 6.14 MPA as the maximum. The average, minimum, and maximum values of the MLPNN-adaboost split tensile strength model vary by 3.65, 49.38, and 18.24%, respectively, as compared to the individual MLPNN model. When compared to the individual split tensile strength MLPNN model, the MLPNN-bagging model exhibits a deflection of 0.91, 61.73, and 18.57 in average, minimum, and maximum values, respectively.

### 6.3. SVM Outcomes

**Figure 16:**
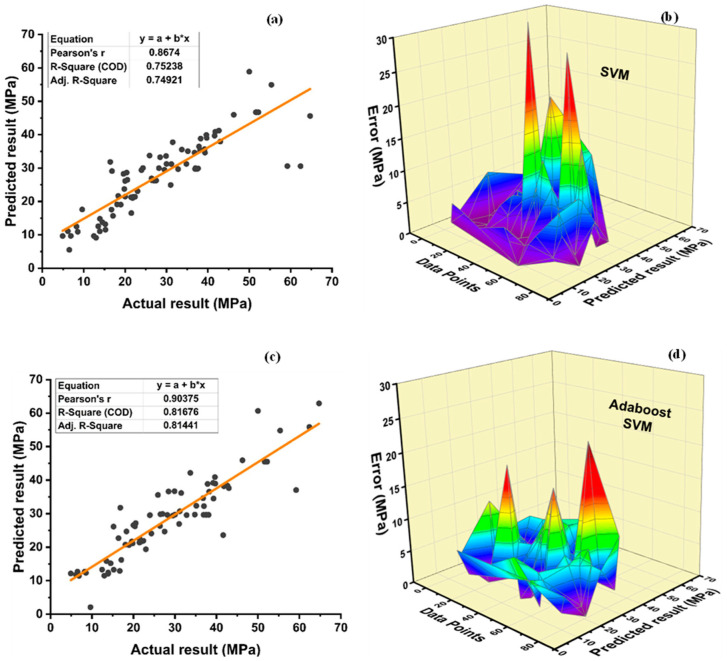

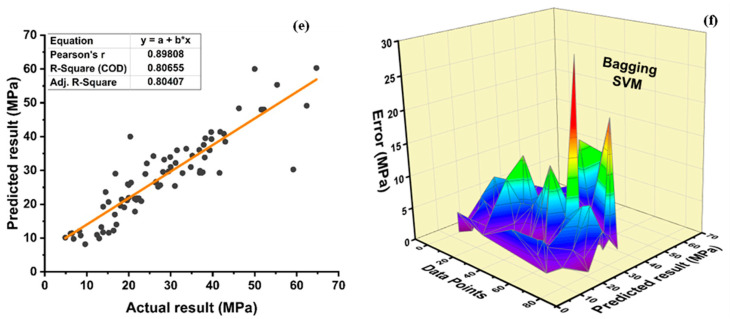
Compressive strength: (**a**) SVM regression model; (**b**) SVM regression model error distribution; (**c**) SVM-Adaboost regression model; (**d**) SVM-Adaboost regression model error distribution; (**e**) SVM-Bagging regression model; (**f**) SVM-Bagging regression model error distribution.

Figure 16 depicts the prediction outcome of f_c_’ with and without ensemble modelling via SVM. It is clear that all supervised machine learning algorithms produce a good correlation between prediction and output. The SVM model yields an R^2^ = 0.752 correlation, as shown in Figure 16a, whereas boosting and bagging yield relationships of approximately 0.817 and 0.807, respectively, as shown in Figure 16c and Figure 16e, respectively. As shown in Figure 16b, an average, maximum, and minimum error of 4.28 MPa, 31.82 MPa, and 0.023 MPa, respectively, for individual SVM model is observed. Furthermore, data illustrates that 70% of the error lies below 5 MPa, 23.75% from 5 to 10 MPa, 1.25% from 10 to 15 MPa, 2.5% from 15 to 20 MPa, and 2.5% above 20 MPa. As shown in Figure 16d, the error distribution of the best f_c_’ SVM ensemble adaboost model has a minimum and maximum error of 0.029 MPa and 22.17 MPa, respectively, with 4.38 MPa as an average error. Moreover, data show that 61.25% of error lies below 5 MPa, 32.5% between 5 and 10 MPa, 3.75% from 10 to 15 MPa, only 1.25% from 15 to 20 MPa, and 20 to 25 MPa, respectively. Figure 16e shows an average, maximum, and minimum error of 4.11 MPa, 28.90 MPa, and 0.0027 MPa, respectively, for the ensemble SVM bagging model for f_c_’. Moreover, bagging ensemble model data depict an error of 72.5% below 5 MPa, 20% from 5 to 10 MPa, 5% from 10 to 15 MPa, 1.25% from 15 to 20 MPa and 1.25% above 20 MPa.

**Figure 17:**
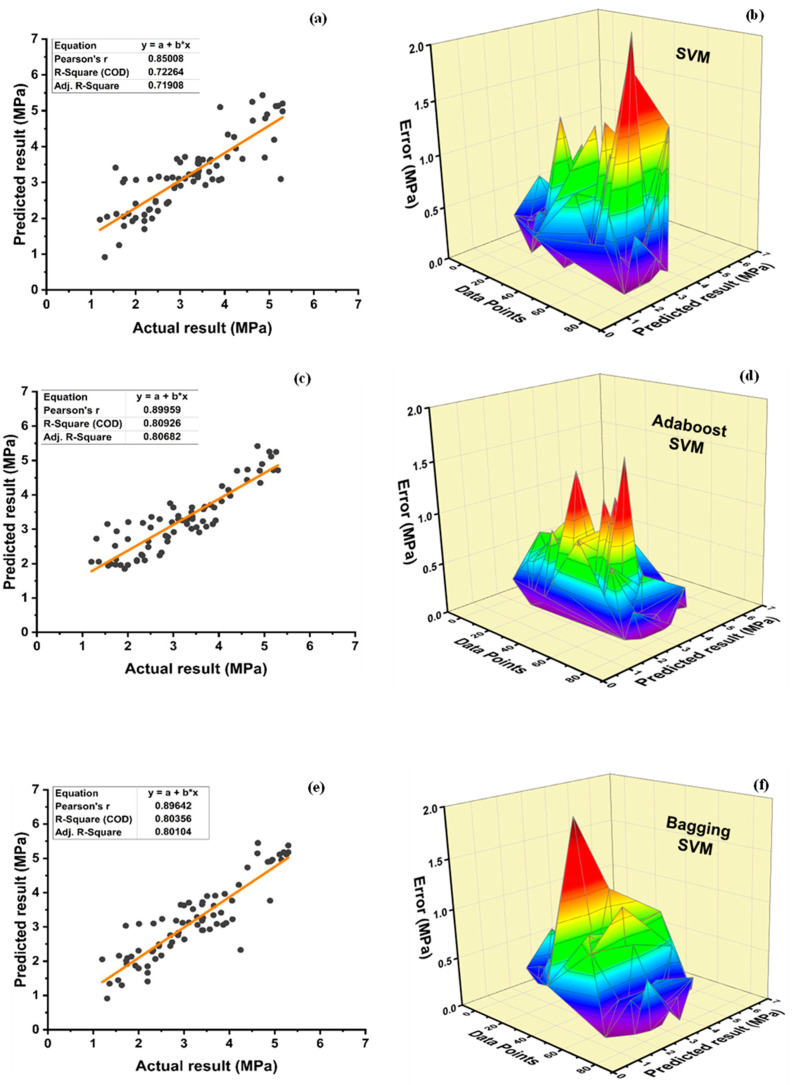
Tensile strength: (**a**) SVM regression model; (**b**) SVM regression model error distribution; (**c**) SVM-Adaboost regression model; (**d**) SVM-Adaboost regression model error distribution; (**e**) SVM-Bagging regression model; (**f**) SVM -Bagging regression model error distribution.

Figure 17a shows R^2^ = 0.723 for individual SVM f_sts_ model. R^2^ value of 0.809 and 0.804 is observed for boosting and bagging ensemble SVM models, respectively, as seen in Figure 17c,e. The average, minimum, and maximum errors observed from Figure 17b are 0.40 MPa, 0.0024 MPa, and 2.167 MPa, respectively, for individual SVM models while 0.35, 1.60, and 0.0097 MPa, respectively, for SVM best f_sts_ ensemble adaboost model, as shown in Figure 17d. Figure 17f illustrates the average, maximum, and minimum error of 0.35, 1.92, and 0.00084 for SVM best ensemble bagging model. An enhancement of 12.5 and 26.16% is observed for average and maximum error values for the SVM best ensemble adaboost model in comparison to the individual SVM f_sts_ model. Similarly, the individual SVM model compared with bagging SVM model depicts an improvement of 12.5, 11.39, and 65% in average, maximum, and minimum errors, respectively. Data of individual SVM model depict an inaccuracy of 91.25% below 1 MPa, 7.5% error between 1 and 2 MPa, and 1.25% error between 2 and 3 MPa. In total, 95% of data of the SVM ensemble models (both bagging and boosting) shows an error of less than 1 MPa, while only 5% of error from 1 to 2 MPa. SVM model can predict the targeted results with an ignorable deviation observed by the value of errors. Moreover, the SVM-boosting ensemble model enhances the R^2^ value by 8.64% and 11.89% for f_c_’ and f_sts_ in comparison to the individual SVM model. Likewise, the SVM bagging ensemble model enhances R^2^ by 7.31% and 11.20% for f_c_’ and f_sts_, respectively. Table 13 and Table 14 illustrate the statistical value of SVM f_c_’ and SVM f_sts_ model, respectively. As illustrated in Table 13, the average, minimum, and maximum compressive strengths obtained from individual SVM models are 27.96 MPa, 5.50 MPa, and 58.89 MPa, respectively. The SVM-adaboost compressive strength model has an average, minimum, and maximum value variation of 2.11, 61.64, and 6.81%, respectively. Similarly, when comparing the SVM bagging model to the individual SVM compressive strength model, the average, minimum, and maximum values diverged by 1.86, 48.73, and 2.43%, respectively. The average result for the SVM split tensile strength model is 3.19 MPa, with the minimum and maximum values being 0.92 MPa and 5.43 MPa, respectively. When compared to individual SVM models, a variance of 2.19%, 0.18%, and 1.01 times is seen in the average, maximum, and minimum values of the SVM-adaboost split tensile strength model. When compared to the individual split tensile strength SVM model, the SVM bagging model exhibits a deflection of 1.57, 1.09, and 0.37% in average, minimum, and maximum values, respectively.

### 6.4. Random Forest Outcomes

**Figure 18:**
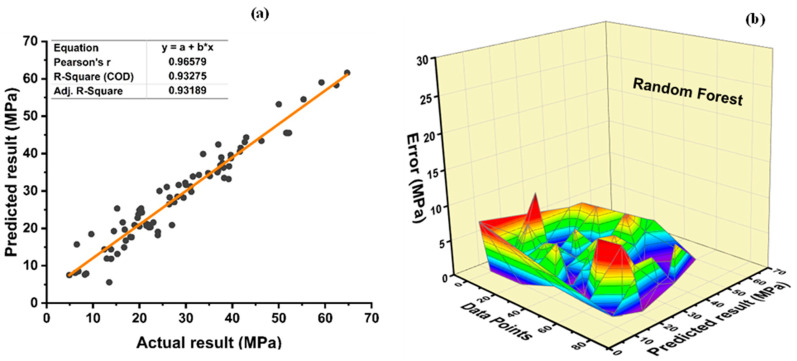
Compressive strength (**a**) RF model; (**b**) RF regression model error distribution.

Random forests are a type of ensemble machine learning technique that combines bagging and random feature selection to generate prediction models. It is an efficient and user-friendly approach. Figure 18 shows the supervised learning approach’s prediction accuracy for f_c_’ of plastic concrete. It is an ensemble model, with a strong correlation to the target values of R^2^ = 0.9327 with an average error distribution, as shown in Figure 18a, demonstrating its precision in the non-linear projection of normal concretes’ strength. Figure 18b demonstrates an average, minimum error of 2.71 MPa and maximum error of 0.0036 MPa, and 10.12 MPa, respectively. Moreover, data show an inaccuracy of 81.25% below 5 MPa, 17.5% between 5 and 10 MPa, and 1.25% between 10 and 15 MPa, respectively. The data show no error above 15 MPa.

**Figure 19:**
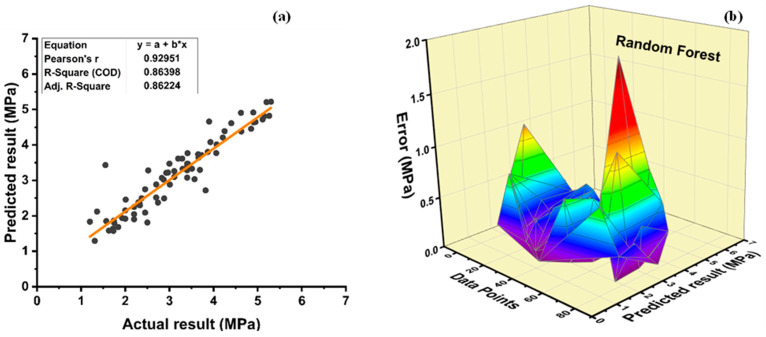
Tensile strength (**a**) RF model; (**b**) RF model error distribution.

Figure 19a shows the RF prediction accuracy of tensile strength of plastic concrete. It is an ensemble with a strong correlation to the target values of R^2^ = 0.8639. Figure 19b shows an average, maximum, and minimum error of 0.27 MPa, 1.88 MPa, and 0.00073 MPa, respectively, for RF model of tensile strength. Moreover, data depict that 96.25% of the error lies below 1 MPa and 3.75% of the error is in between 1 and 2 MPa, respectively.

## 7. Evaluation of Models

**Figure 20:**
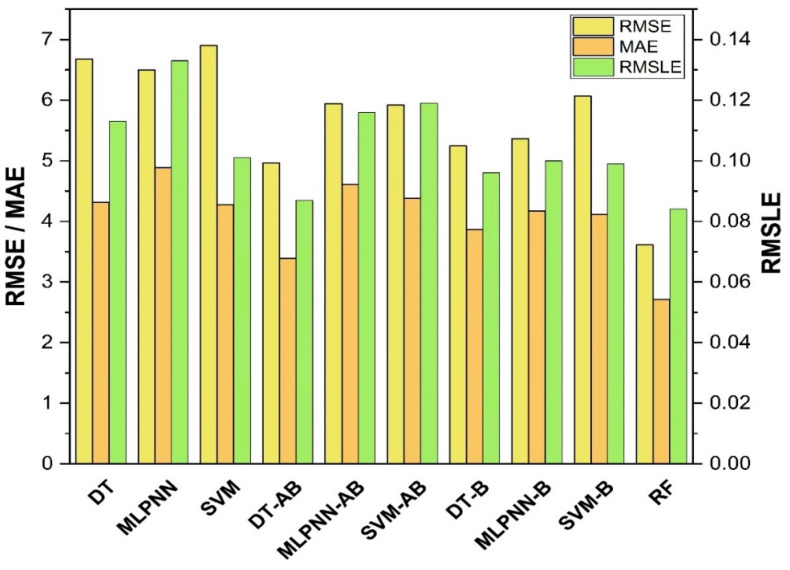
Statistical analysis of compressive strength models.

**Figure 21:**
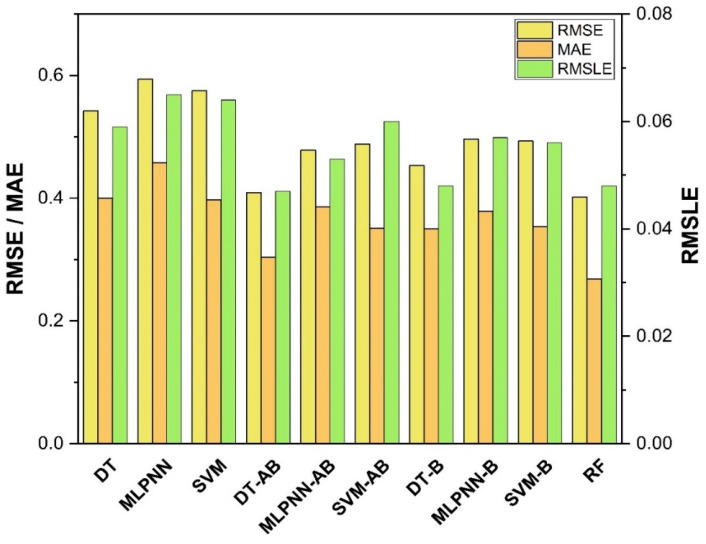
Statistical analysis of tensile strength model.

The results of individual, ensemble bagging, ensemble boosting, and modified ensemble models are evaluated by different errors including correlation coefficient (R^2^), RMSLE, MAE, and RMSE, as shown in Figure 20 for f_c_’ models and Figure 21 and f_sts_ models, and their values are tabulated in Table 15.

A comparison was conducted between the ensemble algorithm and the other individual machine learning algorithms to further demonstrate the ensemble algorithm’s capacity. The model parameter determinations are comparable to ensemble models, for example, setting initial values and then optimizing these values using ensemble algorithm. Ensemble algorithms are widely recognised to contain multiple weak-learners created by individual learning algorithms, weak-learners with excellent performance gain weight, while weak-learners with poor performance lose weight. As a result, it can provide exact projections. As indicated in Table 15, individual learners have higher error-values than ensemble learners with boosting and bagging. This demonstrates that ensemble algorithms besides minimizing the range of error between experimental and predicted results provide precise forecasts.

Among Individual learner models for f_c_’, R^2^ value of 0.780 for MLPNN overrule DT and SVM representing the accuracy of model up to 78%, whereas DT model surpasses the other two model in terms of coefficient of determination (R^2^) for f_sts_. SVM is shown to have the least accurate method in predicting both outcomes. Values of the statistical errors for each method are shown in Table 15.

DT-Adaboost stands out among ensemble learning models with boosting having R^2^ values of 0.875 for f_c_’ model and 0.862 for f_sts_ models, demonstrating the model’s accuracy of about 87%. The prediction accuracy of DT-Adaboost model outperforms the MLPNN model by 26.49%, 16.47%, and 25% for f_c_’ and 21.24%, 14.44%, and 11.32% for f_sts_ in terms of MAE, RMSE, and RMSLE, respectively. Similarly, in terms of MAE, RMSE, and RMSLE, DT-Adaboost model beats SVM model with model accuracy of 22.5%, 16.2%, and 26.89% for f_c_’ and 13.39%, 16.19%, and 21.67% for f_sts_, respectively.

Among ensemble learning models with bagging, DT out-performs the other two methods with R^2^ value of 0.856 for f_c_’ model and 0.829 for f_sts_ model. From evaluation of MAE, RMSE, and RMSLE, again the prediction accuracy of DT-bagging overrule MLPNN by 7.31%, 2.29%, and 4% for f_c_’ and 1.1%, 8.67%, and 15.79% for f_sts_ model, respectively. DT-bagging model tops SVM Bagging models by 6.03%, 13.55%, and 3.03% for f_c_’ and 1.13%, 8.11%, and 14.26% for f_sts_ model in terms of MAE, RMSE, and RMSLE, respectively.

Random forest is classified as modified ensemble learner model, the prediction accuracy of modified ensemble outperforms individual, bagging, and boosting models based on correlation coefficient R^2^ with model accuracy of 93% for f_c_’ model and 86% for f_sts_ model.

Comparing all the models based on the coefficient of determination R^2^ value the prediction accuracy follows the following order, modified ensemble > ensemble-adaboosting > ensemble-Bagging > individual learner.

## 8. Sensitivity Analysis

**Figure 22:**
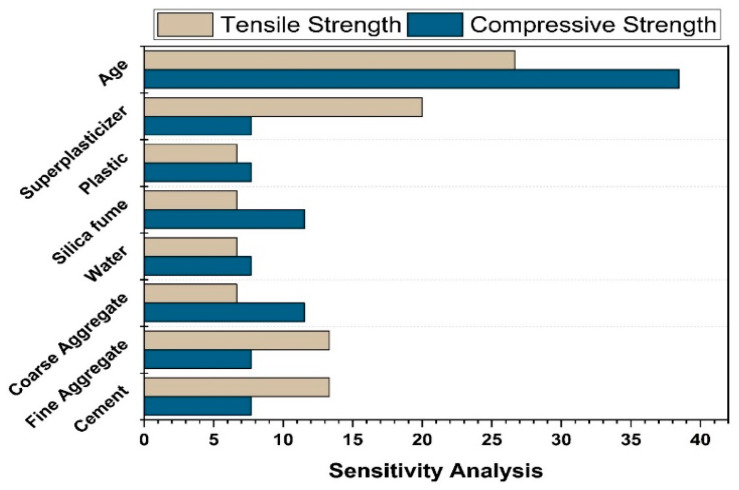
Input parameters’ contribution to compressive and tensile strength models.

Sensitivity analysis is a useful tool for determining the significance of each input to the results. In most real-world applications, identifying all input variables that influence the problem’s results is difficult. Sensitivity analysis is a neural network technology that identifies all important parameters from a list of potential parameters efficiently. After training the neural network on the complete dataset, sensitivity analysis is performed [57]. As input parameters, eight factors were used: cement, water, CA, FA, SP, SF, age, and plastic waste. The influence of every constituent in the production of ensemble models is illustrated in Figure 22. When compared to FA, cement, and other additives, silica fume, coarse aggregate, and age have been demonstrated to contribute more towards compressive strength. Although, for f_sts_ models, CA and water are the least sensitive parameters. The most influential parameters for f_sts_ models include super plasticizer and age. In the creation of both models, FA played a moderate influence.

## 9. K Fold Cross-Validation

Machine learning models’ real performance may be estimated statistically via cross-validation. It is essential to know how the models picked operate. To determine the model data’s correctness, a validation method is needed. To perform the k fold cross-validation test, the dataset must be shuffled and divided into k-groups. In this study, the experimental samples’ data are randomly separated into 10 equal subgroups. Only one of the 10 subsets is utilized for validation, while the other 9 are used to build the model [60].

**Figure 23:**
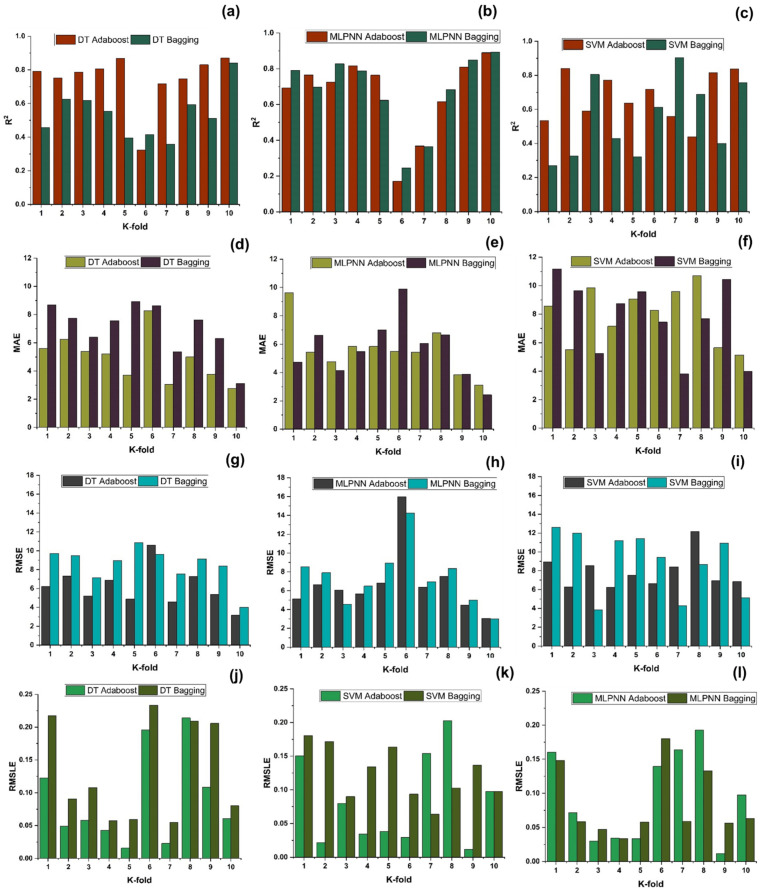
Compressive strength models (**a**–**c**) indicates R^2^ models’ result validated with K fold; (**d**–**f**) indicates MAE models’ result validated with K fold; (**g**–**i**) indicates RMSE models’ result validated with K fold; (**j**–**l**) indicates RMSE models’ result validated with K fold.

**Figure 24:**
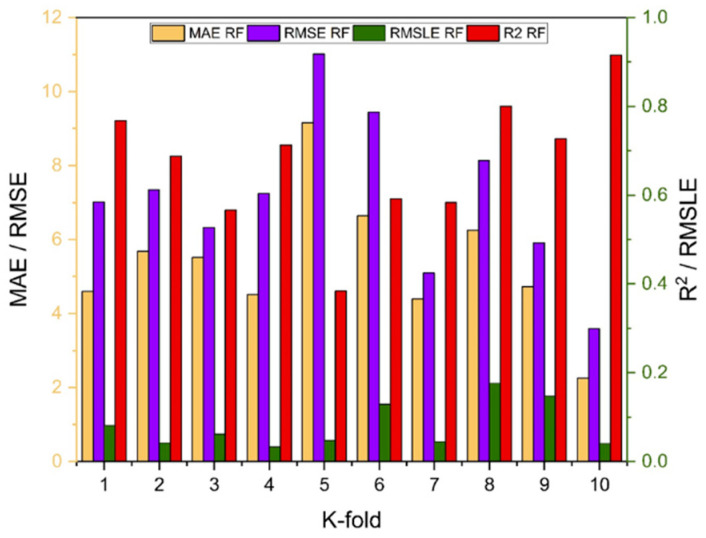
RF models cross validation with different statistical parameters.

**Figure 25:**
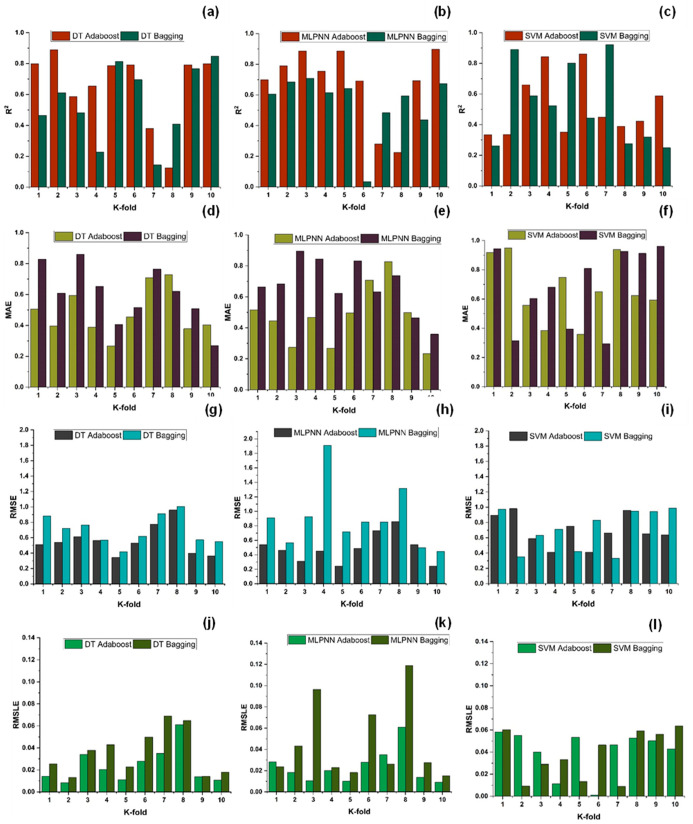
Tensile strength models (**a**–**c**) indicates R^2^ models’ result validated with K fold; (**d**–**f**) indicates MAE models’ result validated with K fold; (**g**–**i**) indicates RMSE models’ result validated with K fold; (**j**–**l**) indicates RMSE models’ result validated with K fold.

**Figure 26:**
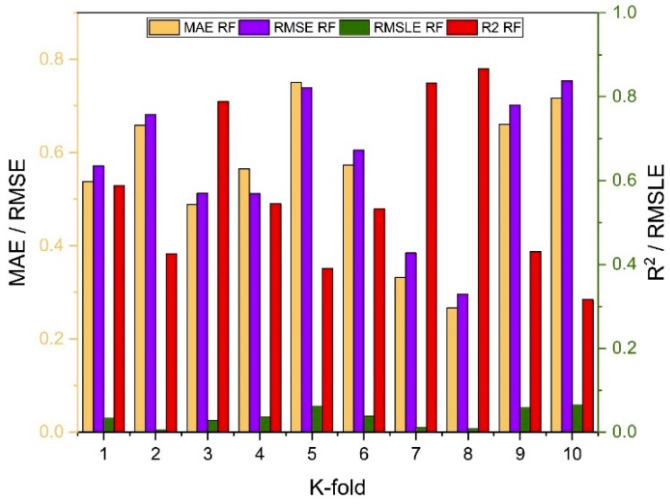
RF models cross validation with different statistical parameters.

As indicated in Figure 23, Figure 24, Figure 25 and Figure 26, the results of cross-validation are expressed as R^2^, MAE, RMSLE, and RMSE. In comparison to supervised machine learning approaches, the RF model has fewer mistakes and a higher R^2^. The average R^2^ value for RF modelling is 0.674 for a tenfold increase in f_c_’, with a minimum value of 0.39 and a maximum value of 0.92, as illustrated in Figure 24. As indicated in Figure 26, the average R^2^ value for f_sts_ is 0.57, with a minimum value of 0.32 and a maximum value of 0.87. Each model exhibits less validation mistakes. The validation indication indicates that the mean values of RMSE, MAE, and RMSLE for the f_c_’ RF model are 5.37, 7.11, and 0.083, respectively, and for f_sts_ RF model are 0.56, 0.58, and 0.035, respectively. Similarly, other ensemble models used in this study exhibit the same tendency, but with far larger errors.

## 10. Conclusions

Experimental tests for compressive strength and tensile strength were performed on 80 cylinders with different proportions for radiated and irradiated plastic for each outcome. Data from the literature were used to train the models containing 320 data points. Testing was performed on the experimental data. Characteristics and mix proportions of the specimens are shown in Section 2.3 and Section 2.4, respectively. Individual and ensemble machine learning models for the prediction of compressive and split tensile strength of plastic concrete are investigated in this paper using DT, MLPNN, SVM, and RF algorithms. The analysis resulted in the following conclusions:

1. The cross-linking effect of gamma radiation on plastic waste was responsible for the material’s improved crystallinity. IPW has shown more crystalline structure showing an increase of 16% in crystallinity as compared to IPW. Hence, enhanced mechanical properties for IPW were observed when compared with RPW specimens;
2. Tensile strength of plastic concrete is marginally affected by high tensile strength of plastic polymer itself, as opposed to its compressive strength;
3. Due to the increased micro-structure, the strength loss was restored by utilizing IPW as a replacement for cement or fine aggregate. It was found that the use of IPW in concrete not only eliminate plastic waste, but also improve the mechanical properties of concrete. Concrete with less cement in it, on the other hand, emits less CO_2_ into the atmosphere;
4. A 1.07% improvement in compressive strength and 13.7% rise in split tensile strength were seen when conventional Portland cement was replaced with IPW. A 5% substitution will minimize 20.20 kg/m^3^ of cement, making the mix environmentally friendly;
5. As compared to the individual technique, ensemble models with boosting and bagging showed better results with fewer errors. The individual models were enhanced using ensemble algorithms. Comparison of different ensemble learning models with boosting and bagging showed that DT performed better with R^2^ of 0.875 and 0.856 for boosting and bagging, respectively, for f_c_’ models and depicted R^2^ value of 0.856 and 0.829 for boosting and bagging, respectively, for f_sts_ models, when compared to MLPNN and SVM;
6. The prediction accuracy of Modified ensemble (RF) outperforms individual, bagging, and boosting models based on correlation coefficient (R^2^) with model accuracy of 93% for f_c_’ model and 86% for f_sts_ model;
7. Sensitivity analyses depicted that FA contributed moderately in the development of the f_c_’ models and f_sts_ models. Moreover, cement, SF, CA, and age played vital role in the development of f_c_’ models. Tensile strength models showed to be affected least by water and CA;
8. K fold validation was utilized for determining the models’ validity R^2^, RMSE, RMSLE, and mean errors. Fewer errors with high correlation were observed. In comparison to supervised machine learning approaches, the RF model has less errors and a higher R^2^. The average R^2^ for RF cross validation is 0.674 for f_c_’ model and 0.57 for f_sts_ model;
9. ML approaches utilized in this study can precisely predict the mechanical properties of concrete. Furthermore, these ML algorithms may be used to create a more sustainable mix design for plastic concrete instead of laborious experimental work demanding a large number of experiments in the laboratory and using a large number of raw materials and manpower.

## 11. Limitations and Direction for Future Work

Compressive and split tensile strengths were calculated with the use of a comprehensive and reliable database. If, however, a more general expression is needed, increasing the database and adding other input parameters may produce the appropriate results. The models established in this paper are for compressive and split tensile strength prediction in plastic concrete. The models predicted the plastic concrete strengths using statistical parameters with high accuracy and reliability. However, by using the same modelling parameters, ANN, ANFIS, and GEP models may be used to forecast the characteristics of concrete that contains a variety of additional concrete ingredients. These models will be modified based on the input parameters, and the predicted outcomes will be heavily influenced by the database used. Additionally, machine learning techniques may be used with heuristic methods such as the whale optimization algorithm, ant colony optimization, and particle swarm optimization to obtain optimal results. These tactics may then be compared to those employed in the current investigation.

## Figures and Tables

**Table 1:** High-density polyethylene (HDPE) properties.

Attribute	Value	Unit
Compressive strength	2.9	ksi
Split tensile strength	4.05	ksi
Impact strength	247.2	kJ/m^2^
Percent elongation	213.1	%
Modulus of elasticity	1.010^3^	MPa
Life of service	>50	years
Thermal conductivity	0.29	kcal/m.hr.c

**Table 2:** Chemical composition of constituents of plastic concrete.

Chemical Composition (Mass % of Oxide)	CaO	SiO_2_	Al_2_O_3_	MgO	SO_3_	TiO_2_	K_2_O	Fe_2_O_3_	Na_2_O	Zn	H_2_O
Binder Type											
OPC	65.2%	19.2%	5.2%	3.4%	1.5%	-	0.62%	2.4%	0.3%	-	-
Silica Fume	0.25	96%	0.25%	0.56%	0.12%		0.56%	0.5%	0.25%	0.02%	0.6%
Regular Plastic	4.87%	64.3%	<0.01%	<0.01%	14.76%	5.0%	1.89%	4.12%	<0.01%	1.7%	-
Irradiated Plastic	5.24%	68.6%	<0.01%	<0.01%	13.47%	5.91%	2.02%	3.26%	<0.01%	0.71%	-

**Table 3:** The range of input and output variables for developing the compressive strength model of plastic concrete.

Parameters	Unit	Abbreviation	Min	Max
Input parameters				
Cement	kg/m^3^	C	100	550
Fine-aggregate	kg/m^3^	FA	80	957
Coarse-aggregate	kg/m^3^	CA	100	1867
Water	kg/m^3^	W	100	238
Silica-fume	kg/m^3^	SF	0	127.9
Plastic	kg/m^3^	P	0	637
Superplasticizer	kg/m^3^	SP	0	8
Age	Days	Age	3	28
Output parameters				
Compressive strength	MPa	f_c_’	2.69	66.89

**Table 4:** The range of input and output variables for developing the split tensile strength model of plastic concrete.

Parameters	Unit	Abbreviation	Min	Max
Input parameters				
Cement	kg/m^3^	C	100	1015
Fine-aggregate	kg/m^3^	FA	80	909
Coarse-aggregate	kg/m^3^	CA	100	1335
Water	kg/m^3^	W	100	260
Silica-fume	kg/m^3^	SF	0	254
Plastic	kg/m^3^	P	0	637
Superplasticizer	kg/m^3^	SP	0	49.5
Age	Days	Age	3	28
Output variable				
Tensile strength	MPa	f_sts_	0.45	8.21

**Table 5:** Data statistical description for compressive strength model’s parameters (kg/m^3^).

Parameters	Cement	Fine Aggregate	Coarse Aggregate	Water	Silica Fume	Plastic	Superplasticizer	Age
Statistical description								
Mean	376.90	642.87	938.56	185.46	8.82	93.77	1.42	20.67
Std error	3.75	9.24	17.21	1.44	1.11	6.79	0.13	0.52
Median	375	671.8	928	191.58	0	25.955	0	28
variance	5639.22	34,182.90	118,510.56	825.83	489.12	18,448.57	6.41	109.24
Std. dev	75.09	184.89	344.25	28.74	22.12	135.83	2.53	10.45
Kurtosis	5.18	1.50	1.19	1.26	12.94	3.17	0.52	−1.40
Skewness	−1.40	−1.22	0.04	−0.89	3.41	1.87	1.47	−0.75
Range	450	877	1767	138	127.9	637	8	25
Min	100	80	100	100	0	0	0	3
Max	550	957	1867	238	127.9	637	8	28
Sum	150,760.35	257,146.50	375,423.84	74,185.07	3528.34	37,509.57	567.45	8267
Count	400	400	400	400	400	400	400	400
Training dataset								
Mean	374.01	643.20	933.46	185.24	9.35	94.45	1.48	20.77
Std error	4.20	10.27	19.52	1.61	1.27	7.76	0.14	0.58
Median	375	672.3	928	191.58	0	25.58	0	28
variance	5651.64	33,748.12	121,956.35	834.10	515.71	19,246.67	6.54	108.32
Std. dev	75.18	183.71	349.22	28.88	22.71	138.73	2.56	10.41
Kurtosis	5.40	1.56	1.03	1.34	11.78	3.08	0.32	−1.37
Skewness	−1.51	−1.22	−0.06	−0.93	3.26	1.87	1.40	−0.77
Range	450	877	1767	138	127.9	637	8	25
Min	100	80	100	100	0	0	0	3
Max	550	957	1867	238	127.9	637	8	28
Sum	119,681.85	205,823.42	298,708.42	59,276.27	2991.81	30,224.74	474.4	6647
Count	320	320	320	320	320	320	320	320
Testing Dataset								
Mean	388.48	641.54	958.94	186.36	6.71	91.06	1.16	20.25
Std error	8.28	21.32	36.33	3.17	2.19	13.90	0.27	1.19
Median	375.50	663.32	928.00	191.58	0.00	28.42	0.00	28.00
variance	5490.71	36,368.97	105,570.79	801.85	382.24	15,450.08	5.88	114.11
Std. dev	74.10	190.71	324.92	28.32	19.55	124.30	2.43	10.68
Kurtosis	4.59	1.46	2.01	1.00	21.42	3.68	1.72	−1.53
Skewness	−1.02	−1.25	0.57	−0.73	4.28	1.82	1.82	−0.68
Range	450	871.6	1767	138	127.9	609	8	25
Min	100	85.4	100	100	0	0	0	3
Max	550	957	1867	238	127.9	609	8	28
Sum	31,078.5	51,323.08	76,715.42	14,908.8	536.53	7284.826	93.05	1620
Count	80	80	80	80	80	80	80	80

**Table 6:** Data statistical description for tensile strength model’s parameters (kg/m^3^).

Parameters	Cement	Fine Aggregate	Coarse Aggregate	Water	Silica Fume	Plastic	Superplasticizer	Age
Statistical description								
Mean	388.89	674.16	874.46	188.65	11.66	81.67	2.08	21.28
Std error	5.15	8.53	14.20	1.72	1.65	6.75	0.30	0.51
Median	376	702	928	197	0	18.5	0	28
variance	10,589.82	29,114.93	80,600.92	1178.76	1089.41	18,216.78	35.26	102.70
Std. dev	102.91	170.63	283.90	34.33	33.01	134.97	5.94	10.13
Kurtosis	13.67	3.40	0.95	0.46	29.44	4.62	48.80	−1.21
Skewness	1.60	−1.65	−0.80	−0.52	4.90	2.20	6.47	−0.87
Range	915	829	1235	160	254	637	49.5	25
Min	100	80	100	100	0	0	0	3
Max	1015	909	1335	260	254	637	49.5	28
Sum	155,555.73	269,662.81	349,782.84	75,459.18	4662.44	32,669.14	833.80	8513.00
Count	400	400	400	400	400	400	400	400
Training dataset								
Mean	390.42	673.89	872.62	190.16	12.54	82.56	2.25	21.04
Std error	5.95	9.59	15.58	1.89	1.98	7.67	0.36	0.57
Median	376	702	928	197	0	19.2	0	28
variance	11,329.68	29,404.05	77,697.30	1142.59	1259.42	18,800.92	42.22	104.58
Std. dev	106.44	171.48	278.74	33.80	35.49	137.12	6.50	10.23
Kurtosis	14.41	3.10	1.09	0.60	26.55	4.60	41.67	−1.31
Skewness	1.99	−1.59	−0.81	−0.58	4.72	2.21	6.09	−0.81
Range	915	829	1235	160	254	637	49.5	25
Min	100	80	100	100	0	0	0	3
Max	1015	909	1335	260	254	637	49.5	28
Sum	124,933.32	215,644.78	279,238.98	60,852.38	4013.86	26,420.02	721.45	6734.00
Count	320	320	320	320	320	320	320	320
Testing Dataset								
Mean	382.78	675.23	881.80	182.59	8.11	78.11	1.40	22.24
Std error	9.80	18.81	34.15	4.02	2.24	14.17	0.30	1.09
Median	376	700	928	191.58	0	15.05	0	28
variance	7689.11	28,314.56	93,277.73	1293.20	400.80	16,072.59	7.00	95.25
Std. dev	87.69	168.27	305.41	35.96	20.02	126.78	2.64	9.76
Kurtosis	3.52	5.11	0.58	0.22	18.19	4.84	0.86	−0.69
Skewness	−1.39	−1.96	−0.81	−0.28	3.87	2.16	1.58	−1.13
Range	450	829	1235	160	127.9	618	8	25
Min	100	80	100	100	0	0	0	3
Max	550	909	1335	260	127.9	618	8	28
Sum	30,622.41	54,018.03	70,543.86	14,606.80	648.58	6249.12	112.35	1779.00
Count	80	80	80	80	80	80	80	80

**Table 7:** R^2^ values of models.

Approaches Employed	Output Parameter	Machine Learning Methods	Ensemble Models	Optimum Estimator	R Value
Individual algorithm	Compressive Strength	DT	-	-	0.779
		MLPNN	-	-	0.780
		SVM	-	-	0.752
	Tensile Strength	DT	-	-	0.767
		MLPNN	-	-	0.736
		SVM	-	-	0.723
Ensemble boosting	Compressive strength	DT-Adaboost	(10, 20, 30…200)	150	0.875
		MLPNN-Adaboost	(10, 20, 30…200)	10	0.814
		SVM-Adaboost	(10, 20, 30…200)	120	0.817
	Tensile Strength	DT-Adaboost	(10, 20, 30…200)	30	0.862
		MLPNN-Adaboost	(10, 20, 30…200)	140	0.814
		SVM-Adaboost	(10, 20, 30…200)	150	0.809
Ensemble bagging	Compressive strength	DT-Bagging	(10, 20, 30…200)	180	0.856
		MLPNN-Bagging	(10, 20, 30…200)	80	0.850
		SVM-Bagging	(10, 20, 30…200)	30	0.807
	Tensile Strength	DT-Bagging	(10, 20, 30…200)	10	0.829
		MLPNN-Bagging	(10, 20, 30…200)	110	0.803
		SVM-Bagging	(10, 20, 30…200)	130	0.804
Modified learner	Compressive strength	RF	(10, 20, 30…200)	20	0.932
Modified learner	Tensile Strength	RF	(10, 20, 30…200)	130	0.864

**Table 8:** Error ranges associated with statistical factors.

Evaluation Criteria	Range	Model Accuracy
MAE	[0, ∞)	Smaller value > better result
RMSE	[0, ∞)	Smaller value > better result
MSLE	[0, ∞)	Smaller value > better result
R^2^ value	(0, 1]	Larger value > better result

**Table 9:** Statistical analysis of compressive strength decision tree model.

Statistical Analysis	DT	DT-Adaboost	DT-Bagging
Average	28.37	27.98	28.50
Minimum	7.59	7.45	7.98
Maximum	64.09	59.43	59.79
No of entries below 10 MPa	3	6	5
No of entries from 10 to 20 MPa	22	15	17
No of entries from 20 to 30 MPa	21	23	20
No of entries from 30 to 40 MPa	14	26	25
No of entries from 40 to 50 MPa	14	5	8
No of entries from 50 to 60 MPa	2	5	5
No of entries from 60 to 70 MPa	4	0	0
Total testing data points	80	80	80
Mean below 10 MPa	0.29	0.58	0.51
Mean in range of 10 to 20 MPa	3.89	3.09	3.40
Mean in range of 20 to 30 MPa	6.45	6.90	6.07
Mean in range of 30 to 40 MPa	5.87	11.25	10.67
Mean in range of 40 to 50 MPa	7.53	2.61	4.30
Mean in range of 50 to 60 MPa	1.28	3.56	3.55
Mean in range of 60 to 70 MPa	3.07	0.00	0.00

**Table 10:** Statistical analysis of tensile strength decision tree model.

Statistical Analysis	DT	DT-Adaboost	DT-Bagging
Average	3.14	3.12	3.14
Minimum	1.32	1.44	1.41
Maximum	4.97	4.94	5.55
No. of entries below 1 MPa	0	0	0
No. of entries from 1 to 2 MPa	20	15	16
No. of entries from 2 to 3 MPa	13	18	20
No. of entries from 3 to 4 MPa	26	30	28
No. of entries from 4 to 5 MPa	21	17	11
No. of entries from 5 to 6 MPa	0	0	5
No. of entries from 6 to 7 MPa	0	0	0
Total testing data points	80	80	80
Mean below 1 MPa	0.00	0.00	0.00
Mean in range of 1 to 2 MPa	0.43	0.33	0.35
Mean in range of 2 to 3 MPa	0.41	0.56	0.63
Mean in range of 3 to 4 MPa	1.10	1.26	1.23
Mean in range of 4 to 5 MPa	1.20	0.97	0.60
Mean in range of 5 to 6 MPa	0.00	0.00	0.33
Mean in range of 6 to 7 MPa	0	0	0

**Table 11:** Statistical analysis of compressive strength MLPNN model.

Statistical Analysis	MLPNN	MLPNN-Adaboost	MLPNN-Bagging
Average	28.88	28.31	28.85
Minimum	2.50	3.59	6.66
Maximum	58.23	56.34	57.56
No. of entries below 10 MPa	8	4	3
No. of entries from 10 to 20 MPa	10	16	15
No. of entries from 20 to 30 MPa	24	24	27
No. of entries from 30 to 40 MPa	23	26	21
No. of entries from 40 to 50 MPa	12	5	9
No. of entries from 50 to 60 MPa	3	5	5
No. of entries from 60 to 70 MPa	0	0	0
Total testing data points	80	80	80
Mean below 10 MPa	0.84	0.35	0.32
Mean in range of 10 to 20 MPa	1.67	2.89	2.55
Mean in range of 20 to 30 MPa	7.35	7.31	8.34
Mean in range of 30 to 40 MPa	10.30	11.65	9.44
Mean in range of 40 to 50 MPa	6.56	2.68	4.73
Mean in range of 50 to 60 MPa	2.16	3.43	3.45
Mean in range of 60 to 70 MPa	0.00	0.00	0.00

**Table 12:** Statistical analysis of tensile strength MLPNN model.

Statistical Analysis	MLPNN	MLPNN-Adaboost	MLPNN-Bagging
Average	3.29	3.17	3.26
Minimum	0.81	1.21	1.31
Maximum	6.14	5.02	5.00
No. of entries below 1 MPa	1	0	0
No. of entries from 1 to 2 MPa	7	6	3
No. of entries from 2 to 3 MPa	21	27	29
No. of entries from 3 to 4 MPa	36	30	29
No. of entries from 4 to 5 MPa	6	16	19
No. of entries from 5 to 6 MPa	8	1	0
No. of entries from 6 to 7 MPa	1	0	0
Total testing data points	80	80	80
Mean below 1 MPa	0.01	0.00	0.00
Mean in range of 1 to 2 MPa	0.12	0.13	0.06
Mean in range of 2 to 3 MPa	0.65	0.80	0.88
Mean in range of 3 to 4 MPa	1.57	1.31	1.25
Mean in range of 4 to 5 MPa	0.33	0.87	1.08
Mean in range of 5 to 6 MPa	0.53	0.06	0.00
Mean in range of 6 to 7 MPa	0.08	0.00	0.00
Average	3.29	3.17	3.26
Minimum	0.81	1.21	1.31
Maximum	6.14	5.02	5.00
No. of entries below 1 MPa	1	0	0
No. of entries from 1 to 2 MPa	7	6	3
No. of entries from 2 to 3 MPa	21	27	29
No. of entries from 3 to 4 MPa	36	30	29
No. of entries from 4 to 5 MPa	6	16	19
No. of entries from 5 to 6 MPa	8	1	0
No. of entries from 6 to 7 MPa	1	0	0
Total testing data points	80	80	80
Mean below 1 MPa	0.01	0.00	0.00
Mean in range of 1 to 2 MPa	0.12	0.13	0.06
Mean in range of 2 to 3 MPa	0.65	0.80	0.88
Mean in range of 3 to 4 MPa	1.57	1.31	1.25
Mean in range of 4 to 5 MPa	0.33	0.87	1.08
Mean in range of 5 to 6 MPa	0.53	0.06	0.00
Mean in range of 6 to 7 MPa	0.08	0.00	0.00

**Table 13:** Statistical analysis of compressive strength SVM model.

Statistical Analysis	SVM	SVM-Adaboost	SVM-Bagging
Average	27.96	28.55	28.48
Minimum	5.50	2.11	8.18
Maximum	58.89	62.90	60.32
No. of entries below 10 MPa	5	1	3
No. of entries from 10 to 20 MPa	15	16	16
No. of entries from 20 to30 MPa	27	34	29
No. of entries from 30 to 40 MPa	24	19	20
No. of entries from 40 to 50 MPa	7	6	9
No. of entries from 50 to 60 MPa	2	2	1
No. of entries from 60 to 70 MPa	0	2	2
Total testing data points	80	80	80
Mean below 10 MPa	0.54	0.03	0.35
Mean in range of 10 to 20 MPa	2.71	2.69	2.78
Mean in range of 20 to 30 MPa	8.90	11.06	9.33
Mean in range of 30 to 40 MPa	10.46	8.53	8.76
Mean in range of 40 to 50 MPa	3.92	3.32	5.07
Mean in range of 50 to 60 MPa	1.42	1.38	0.69
Mean in range of 60 to 70 MPa	0.00	1.54	1.50

**Table 14:** Statistical analysis of tensile strength SVM model.

Statistical Analysis	SVM	SVM-Adaboost	SVM-Bagging
Average	3.19	3.26	3.14
Minimum	0.92	1.85	0.91
Maximum	5.43	5.42	5.45
No. of entries below 1 MPa	1	0	1
No. of entries from 1 to 2 MPa	7	6	9
No. of entries from 2 to 3 MPa	20	23	24
No. of entries from 3 to 4 MPa	39	36	33
No. of entries from 4 to 5 MPa	7	11	6
No. of entries from 5 to 6 MPa	6	4	7
No. of entries from 6 to 7 MPa	0	0	0
Total testing data points	80	80	80
Mean below 1 MPa	0.01	0.00	0.01
Mean in range of 1 to 2 MPa	0.16	0.15	0.18
Mean in range of 2 to 3 MPa	0.60	0.71	0.73
Mean in range of 3 to 4 MPa	1.63	1.52	1.40
Mean in range of 4 to 5 MPa	0.40	0.63	0.36
Mean in range of 5 to 6 MPa	0.39	0.26	0.46
Mean in range of 6 to 7 MPa	0.00	0.00	0.00

**Table 15:** Statistical errors for the models.

Approach Used	Output Parameter	ML Methods	MAE	RMSE	RMSLE	R^2^
Individual Learner	Compressive Strength	DT	4.317	6.678	0.113	0.779
		MLPNN	4.887	6.501	0.133	0.780
		SVM	4.276	6.902	0.101	0.752
	Tensile Strength	DT	0.400	0.542	0.059	0.767
		MLPNN	0.458	0.594	0.065	0.737
		SVM	0.397	0.575	0.064	0.723
Ensemble Learning Boosting	Compressive Strength	DT-Adaboost	3.391	4.962	0.087	0.875
		MLPNN-Adaboost	4.613	5.940	0.116	0.814
		SVM-Adaboost	4.376	5.921	0.119	0.817
	Tensile Strength	DT-Adaboost	0.304	0.409	0.047	0.862
		MLPNN-Adaboost	0.386	0.478	0.053	0.814
		SVM-Adaboost	0.351	0.488	0.060	0.809
Ensemble Learning Bagging	Compressive Strength	DT-Bagging	3.865	5.244	0.096	0.856
		MLPNN-Bagging	4.170	5.367	0.100	0.850
		SVM-Bagging	4.113	6.066	0.099	0.807
	Tensile Strength	DT-Bagging	0.350	0.453	0.048	0.829
		MLPNN-Bagging	0.379	0.496	0.057	0.803
		SVM-Bagging	0.354	0.493	0.056	0.804
Modified Ensemble	Compressive Strength	Random Forest	2.712	3.613	0.084	0.933
	Tensile Strength	Random Forest	0.268	0.402	0.048	0.864

## Data Availability

Not applicable.
